# A generalized Hosmer–Lemeshow goodness-of-fit test for a family of generalized linear models

**DOI:** 10.1007/s11749-023-00912-8

**Published:** 2023-12-19

**Authors:** Nikola Surjanovic, Richard A. Lockhart, Thomas M. Loughin

**Affiliations:** 1 Department of Statistics, University of British Columbia, Vancouver, BC Canada; 2 Department of Statistics and Actuarial Science, Simon Fraser University, Burnaby, BC Canada

**Keywords:** Empirical regression process, Exponential dispersion family, Generalized linear model, Goodness-of-fit test, Hosmer–Lemeshow test, 62J12, 62E20, 62P99

## Abstract

Generalized linear models (GLMs) are very widely used, but formal goodness-of-fit (GOF) tests for the overall fit of the model seem to be in wide use only for certain classes of GLMs. We develop and apply a new goodness-of-fit test, similar to the well-known and commonly used Hosmer–Lemeshow (HL) test, that can be used with a wide variety of GLMs. The test statistic is a variant of the HL statistic, but we rigorously derive an asymptotically correct sampling distribution using methods of Stute and Zhu (Scand J Stat 29(3):535–545, 2002) and demonstrate its consistency. We compare the performance of our new test with other GOF tests for GLMs, including a naive direct application of the HL test to the Poisson problem. Our test provides competitive or comparable power in various simulation settings and we identify a situation where a naive version of the test fails to hold its size. Our generalized HL test is straightforward to implement and interpret and an R package is publicly available.

**Supplementary Information:**

The online version contains supplementary material available at 10.1007/s11749-023-00912-8.

## Introduction

Generalized linear models (GLMs), including among others the linear, logistic, and Poisson regression models, have been used in a vast number of application domains and are extremely popular in medical and biological applications.

Naturally, it is desirable to have a model that fits the observed data well. Hosmer and Lemeshow (1980) constructed a GOF test for the logistic regression model, the HL test, which applies a Pearson test statistic to differences of observed and expected event counts from data grouped based on the ordered fitted values from the model. As a result, their test is very easy to interpret and is extremely popular, particularly in medical applications. Due to the simplicity of the test, it is tempting to naively apply it to other GLMs with minimal modification, as has occasionally been suggested in the literature (Bilder and Loughin 2014; Agresti 1996, p. 90). However, the HL test has not been rigorously justified outside of the binomial setting, and hence, its validity is unknown. Indeed, there are indications that its limiting distribution is incorrect in the non-binomial setting, as simulation results in Sect. 5 suggest.

A considerable number of GOF tests available for GLMs with a non-binomial or non-normal response (or other regression models beyond GLMs) involve kernel density estimation or some other form of smoothing—for example, the test of Cheng and Wu (1994). However, González-Manteiga and Crujeiras (2013) mention that the selection of the smoothing parameter used in some tests is “a broadly studied problem in regression estimation but with serious gaps for testing problems”. Also, use of continuous covariates in a GLM model can render certain basic tests, such as the Pearson chi-squared test, invalid (Pulkstenis and Robinson 2002).

In this paper, we work towards two goals. First, we explore some of the properties of the “naive” application of the HL test to other GLM distributions. Second, we derive a more appropriate modification to the HL test statistic and determine its correct limiting null sampling distribution. The modification is based on an application of theory developed by Stute and Zhu (2002). We show that the test statistic has an asymptotic chi-squared distribution for many GLMs in the exponential dispersion family, adding to the appealing simplicity of the test. We investigate both the new and the naive tests’ small-sample performances in a series of carefully designed computational experiments.

Section 2 gives an overview of previously developed GOF tests. Section 3 has a more detailed description of certain GOF tests, including the naive HL test and our new test. We present our main theorems concerning the asymptotic distribution of our proposed test under the null hypothesis as well as consistency results. The design for our simulation study comparing these tests is laid out in Sect. 4, and the results are provided in Sect. 5. We find that our test provides competitive or comparable power to other available tests in various simulation settings, is computationally efficient, and avoids the use of kernel-based estimators. Finally, we discuss the results and potential future work in Sect. 6. Proofs of several results are in the supplementary material and an R package implementing the generalized Hosmer–Lemeshow test is available at https://github.com/nikola-sur/goodGLM.

## Background and notation

We let *Y* be a response variable that is associated with a covariate vector, *X*, where $X \in {\mathbb {R}}^d$. We write $(X_i, Y_i)$, $i=1,\ldots ,n$, to denote a random sample where each $(X_i, Y_i)$ has the same distribution as (*X*, *Y*), and provides observed data $(x_i,y_i)$. Limits are taken with the sample size *n* tending to infinity.

The HL GOF test assesses departures between observed and expected event counts from data grouped based on the fitted values from the GLM. For instance, in the binary case for logistic regression, we assume

$$\begin{aligned} {{\,\textrm{E}\,}}(Y \mid X=x) = \pi (\beta ^\top x) = \frac{\exp (\beta ^\top x)}{1+\exp (\beta ^\top x)}, \end{aligned}$$

for some $\beta \in {\mathbb {R}}^d$. The likelihood function is then given by

$$\begin{aligned} {\mathcal {L}}(\beta ) = \prod _{i=1}^n \pi (\beta ^\top x_i)^{y_i} (1-\pi (\beta ^\top x_i))^{1-y_i}, \end{aligned}$$

from which a maximum likelihood estimate (MLE), $\beta _n$, of $\beta$ can be obtained. Computing the HL test statistic starts with partitioning the data into *G* groups. This is often done in a way so that the groups are of approximately equal size and fitted values within each group are similar. The partition fixes interval endpoints, $k_g$ for $1 \le g \le G$, and $-\infty = k_0< k_1< \cdots< k_{G-1} < k_G = \infty$. The $k_g$ are often set to be equal to the logit of equally spaced quantiles of the fitted values, ${\hat{\pi _i}} = \pi (\beta _n^\top x_i)$. We define $I_i^{(g)} = \mathbbm {1}(k_{g-1} < \beta _n^\top x_i \le k_g)$, $O_g = \sum _{i=1}^n y_i I_i^{(g)}$, $E_g = \sum _{i=1}^n {\hat{\pi _i}} I_i^{(g)}$, $n_g = \sum _{i=1}^n I_i^{(g)}$, and ${\bar{\pi }}_g = E_g/n_g$ for $1 \le g \le G$, where $\mathbbm {1}(A)$ is the indicator function on a set *A*. Here, $n_g$ represents the number of observations in the *g*th group, and ${\bar{\pi }}_g$ represents the average of the fitted values within the *g*th group. The HL test statistic is

1 $$\begin{aligned} {\widehat{C}}_G = \sum _{g=1}^G \frac{(O_g - E_g)^2}{n_g {\bar{\pi }}_g (1-{\bar{\pi }}_g)}. \end{aligned}$$

The theory behind this test, based on work by Moore and Spruill (1975), suggests that the asymptotic null distribution of the test statistic follows the distribution of a weighted sum of chi-squared random variables. Hosmer and Lemeshow (1980) *approximated* this distribution with a single chi-squared distribution, where the $G-2$ degrees of freedom were determined partly by simulation.

To generalize the HL test to other GLMs, we follow Stute and Zhu (2002). We assume that ${{\,\textrm{E}\,}}(Y^2) < \infty$ under the null hypothesis, given by (4) below. As a consequence, ${{\,\textrm{E}\,}}(Y)<\infty$, and we may define $m^*(x) = {{\,\textrm{E}\,}}(Y \mid X = x)$, and $\sigma ^2(x) ={{\,\textrm{Var}\,}}(Y \mid X=x)$. We also assume that $\sigma ^2(x)>0$ for all *x* in the support of *X*. The GLM that we fit and test assumes that the conditional density of *Y* given $X=x$ is an exponential family member with inverse link function, *m*. Specifically,

2 $$\begin{aligned} f_{Y\mid X}(y\mid x,\beta _0) = \exp \{y\theta - b(\theta )\} \nu (dy), \end{aligned}$$

where the vector $\beta _0$, $\theta$, and *x* are related by ${{\,\textrm{E}\,}}(Y\mid X=x)=b'(\theta )=m(\beta _0^\top x)$, and $\nu$ is a suitable dominating measure. The parameter $\theta$ must belong to $\Theta =\{\theta : \int \exp (y\theta )\nu (dy)<\infty \}$ and we must have $m(\beta _0^\top x) \in b'(\Theta )$ for all *x* in the support of *X*. In such a model, the variance is a function of the mean and we may write $\sigma ^2(x) = v(m(\beta _0^\top x))$, for a smooth function *v* determined by the function *b*. In some cases it is of interest to add an (unknown) dispersion parameter, $\phi$, to the model and assume further that there is a scalar $\phi _0$ such that the density of *Y* given $X=x$ has the form

3 $$\begin{aligned} f_{Y\mid X}(y\mid x,\beta _0,\phi _0) = \exp \left\{ \frac{y\theta - b(\theta )}{\phi _0} - c(y,\phi _0)\right\} \nu (dy), \end{aligned}$$

where $\nu$ is a (possibly different) dominating measure, and $\beta _0$, $\theta$, and *x* are related as before. With $f_{Y\mid X}$ as in (3), we can write $\sigma ^2(x) = \phi _0 \cdot v(m(\beta _0^\top x))$. The parameter space for $(\theta ,\phi )$ is $\Theta \otimes \Lambda$ for a suitable $\Lambda \subset (0,\infty )$.

Let ${\textbf{B}}_0 =\{\beta :P(m(\beta ^\top X) \in b'(\Theta ))=1\}$. We test the null hypothesis

4 $$\begin{aligned} H_0: {\left\{ \begin{array}{ll} \begin{aligned} &{}\exists \beta \in {\textbf{B}}_0 \text { s.t. } m^*(X) = m(\beta ^\top X) \text { a.s., and} \\ &{} P(X \in \{x \text { s.t. } Y \mid X = x \sim f_{Y\mid X = x}\}) = 1, \end{aligned} \end{array}\right. } \end{aligned}$$

for a pre-specified inverse link function $m(\cdot )$. Our alternative hypothesis is

5 $$\begin{aligned} H_1: {\left\{ \begin{array}{ll} \begin{aligned} \forall \beta &{}\in {\textbf{B}}_0, \text {we have } P\left( m^*(X) \ne m(\beta ^\top X)\right)>0 \text { or} \\ P&{}\left( X\in \{ x \text { s.t. } Y\mid X=x \not \sim f_{Y\mid X=x}\}\right) >0. \end{aligned} \end{array}\right. } \end{aligned}$$

That is, we simultaneously test for a misspecification of the link function and of the conditional response distribution. We denote the maximum likelihood estimate of $\beta$ by $\beta _n$ and let $\phi _n$ be some consistent estimate of $\phi$; if there is no dispersion parameter, we take $\phi _n=\phi _0=1$.

For many popular link functions $m^{-1}$, it is automatic that $m(\beta ^\top X) \in b'(\Theta )$ almost surely for all $\beta \in {\mathbb {R}}^d$ and any *X* distribution. For other link functions and some distributions for the predictor *X* this may not hold for all $\beta$; this motivates our definition of ${\textbf{B}}_0$. Similar comments apply to models with an unknown dispersion parameter as in (3); see the supplementary material.

**Related work**

A grouped GOF test for logistic regression models similar to the HL test was introduced by Tsiatis (1980), which uses a *data-independent* partitioning scheme (i.e., not random). Canary et al. (2016) constructed a generalized Tsiatis test statistic for binary regression models with a non-canonical link, and then used a data-dependent partitioning scheme. We note that the theoretical validity of such a data-dependent partitioning method for the generalized Tsiatis test should be verified, similar to what was done by Halteman (1980) for logistic regression. Our work focuses on proving the validity of a large class of data-dependent partitioning schemes, also allowing for extensions to a much wider class of GLMs, as Canary (2013) briefly suggested. Our test also extends the $X^2_w$ statistic with all weights equal to one, from Hosmer and Hjort (2002), to a broader class of problems. There are some other versions of the HL test for specific models other than logistic regression, including binomial regression models with a log link (Blizzard and Hosmer 2006; Quinn et al. 2015) and other non-canonical links (Canary et al. 2016). Other models include the multinomial regression model (Fagerland et al. 2008) and the proportional odds and other ordinal logistic regression models (Fagerland and Hosmer 2013, 2016).

GOF tests not based on the HL test have been constructed that can be used with broader classes of GLM models (Su and Wei 1991; Stute and Zhu 2002; Cheng and Wu 1994; Lin et al. 2002; Liu et al. 2004; Rodríguez-Campos et al. 1998; Xiang and Wahba 1995). A review of GOF tests for regression models is given by González-Manteiga and Crujeiras (2013). While many of these tests appear to have merit, they do not seem to have been widely adopted in practice. For instance, the p-values accompanying some tests need to be obtained through simulation and calculations may be time consuming in the presence of several explanatory variables (Christensen and Lin 2015).

## Methods and test statistics

Tests for GOF require statistics measuring departures from the null hypothesis. We use the residual process defined in Stute and Zhu (2002) for $u \in {\mathbb {R}}$ by:

6 $$\begin{aligned} R_n^1(u) = \frac{1}{\sqrt{n}} \sum _{i=1}^n \mathbbm {1}(\beta _n^\top X_i \le u) [Y_i - m(\beta _n^\top X_i)]. \end{aligned}$$

For the special case of logistic regression, the HL test can be rewritten as a quadratic form, in terms of $R_n^1(u)$. Define the (length *G*) vector

7 $$\begin{aligned} S_n^1 = (R_n^1(k_1) - R_n^1(k_0), \ldots , R_n^1(k_G) - R_n^1(k_{G-1}) )^\top . \end{aligned}$$

Then, the HL test statistic can be rewritten as ${\widehat{C}}_G = S_n^{1 \top } D^{-1} S_n^1$, where

8 $$\begin{aligned} D = \text {diag}\left( \frac{n_g {\bar{\pi }}_g(1-{\bar{\pi }}_g)}{n}\right) , \quad 1 \le g \le G. \end{aligned}$$

Provided that $G>d$—a requirement not always cited in references to the HL test—and using Theorem 5.1 in Moore and Spruill (1975), Hosmer and Lemeshow (1980) show that their test statistic is asymptotically distributed under the null hypothesis as a weighted sum of chi-squared random variables, with

$$\begin{aligned} {\widehat{C}}_G \xrightarrow {d} \chi ^2_{G-d} + \sum _{j=1}^d \lambda _j \chi _{1j}^2, \end{aligned}$$

where each $\chi _{1j}^2$ is a chi-squared random variable with 1 degree of freedom, and each $\lambda _j$ is an eigenvalue of a particular matrix that depends on $\beta _0$ and the distribution of *X*. Then, through simulations, they conclude that the term $\sum _{j=1}^d \lambda _d \chi ^2_{1j}$ can be approximated in various settings by a $\chi ^2_{d-2}$ distribution, leading to the recommended $G-2$ degrees of freedom. In other words, ${\widehat{C}}_G {\mathop {\sim }\limits ^{.}} \chi ^2_{G-2}.$ However, in certain settings with a finite sample size this does not serve as a good approximation, as we discuss in Sect. 5.

### Naive generalization of the Hosmer–Lemeshow test

The HL test statistic depends on the binomial assumption only through *D* in (8), with the *g*th diagonal element representing an estimate of the variance of the counts in the *g*th group, divided by *n*. To extend this test to other GLMs, it is tempting to define a “naive” HL test statistic

9 $$\begin{aligned} {\widehat{C}}^*_G = S_n^{1 \top } (D^*)^{-1} S_n^1, \end{aligned}$$

where

$$\begin{aligned} D^* = {{\,\textrm{diag}\,}}\left( \frac{1}{n} \sum _{i=1}^n {\widehat{{{\,\textrm{Var}\,}}}}(Y\mid X=x_i) \mathbbm {1}(k_{g-1} < \beta _n^\top x_i \le k_g) \right) , \end{aligned}$$

for $1 \le g \le G$, similar to the estimates of the variances of group counts given in the original HL test through *D*. For example, for Poisson regression models,

$$\begin{aligned} D^* = {{\,\textrm{diag}\,}}\left( \frac{1}{n} \sum _{i=1}^n m(\beta _n^\top x_i) \mathbbm {1}(k_{g-1} < \beta _n^\top x_i \le k_g) \right) , \end{aligned}$$

since the conditional variance of the response is equal to the conditional mean. This idea is very briefly suggested in Agresti (1996) on p. 90, and in Bilder and Loughin (2014), but has not been developed further or assessed in the literature. The limiting distribution of this test statistic has not been determined, although one might naively assume that it retains the same $\chi ^2_{G-2}$ limit as the original HL test. Implementing this test for Poisson regression models, our simulation results suggest that as the number of estimated parameters in the model increases, the mean and variance of the ${\widehat{C}}^*_G$ test statistic tend to decrease for a fixed sample size. Thus, it is apparent that the naive limiting distribution is not correct. Further properties of this test are discussed in Sect. 5.

### The generalized HL test statistic

The HL test uses a limiting distribution that is only partially supported mathematically and does not seem to be appropriate for finite samples for GLMs outside of logistic regression. Rather than try to fix the flaws in the naive HL test, we propose a test statistic whose limiting law is demonstrated by appropriate techniques to be chi-squared with a determined number of degrees of freedom (less than or equal to *G*). We allow cell boundaries $k_{n,g}$ that may depend on the data but must be distinct and properly ordered for each *n*. We assume each $k_{n,g}$ converges in probability to some $k_g$, that these limits are all distinct and that they satisfy $P(\beta _0^\top X = k_g) = 0$ for all *g*. In our simulation study, described in Sect. 4, we use random interval endpoints so that $\sum _{i=1}^n {\hat{\sigma }}^2(x_i) I_i^{(g)}$ is approximately equal across groups. Implementation details are in the supplementary material.

We follow Stute and Zhu (2002) to directly develop our generalized HL test and rigorously determine its correct limiting distribution. We first define

$$\begin{aligned} \left( G_n\right) _{gi}&= \mathbbm {1}(k_{n,g-1} < \beta _n^\top x_i \le k_{n,g}), \\ V^{1/2}&= {{\,\textrm{diag}\,}}\left( \left[ v(m(\beta ^\top x_i))\right] ^{1/2} \right) \big |_{\beta = \beta _0}, \\ W^{1/2}&= {{\,\textrm{diag}\,}}\left( \frac{m'(\beta ^\top x_i)}{v(m(\beta ^\top x_i))^{1/2}} \right) \Biggr |_{\beta = \beta _0}, \end{aligned}$$

for $i = 1, \ldots , n$, and $g = 1, \ldots , G$. Note that in the above definition of $W^{1/2}$, we write $m'(u)$ to denote the derivative of *m*(*u*) with respect to *u*. Also, let $X^*$ be the $n \times d$ matrix whose *i*th row is given by $x_i^\top$, for $i=1,\ldots ,n$. Define $W_n^{1/2}$ and $V_n^{1/2}$ the same as $W^{1/2}$ and $V^{1/2}$, respectively, but evaluated at $\beta _n$ instead of $\beta _0$.

Let $I_n$ be the $n \times n$ identity matrix, and denote the generalized hat matrix by $H_n = W_n^{1/2} X^* (X^{*\top } W_n X^*)^{-1} X^{*\top } W_n^{1/2}$. Define

10 $$\begin{aligned} \begin{aligned} \Sigma _n&= \frac{1}{n} G_n \left( V_n - V_n^{1/2} W_n^{1/2} X^* (X^{*\top } W_n X^*)^{-1} X^{*\top } W_n^{1/2} V_n^{1/2}\right) G_n^{\top } \\&= \frac{1}{n} G_n V_n^{1/2} \left( I_n - W_n^{1/2} X^* (X^{*\top } W_n X^*)^{-1} X^{*\top } W_n^{1/2}\right) V_n^{1/2} G_n^{\top } \\&= \frac{1}{n} G_n V_n^{1/2} (I_n-H_n) V_n^{1/2} G_n^{\top }. \end{aligned} \end{aligned}$$

Denote the Moore–Penrose pseudoinverse of a matrix *A* by $A^+$. Our “generalized HL” (GHL) test statistic is then given as

11 $$\begin{aligned} X^2_{\text {GHL}} = S_n^{1\top } \Sigma _n^{+} S_n^1 /\phi _n. \end{aligned}$$

We note that without the $H_n$ term in $\Sigma _n$, the GHL test statistic reduces to the naive GHL statistic. Under some conditions described next, we have under the null hypothesis that

12 $$\begin{aligned} S_n^{1\top } \Sigma _n^{+} S_n^1/\phi _n \xrightarrow {d} \chi ^2_r, \end{aligned}$$

with $r = {{\,\textrm{rank}\,}}(\Sigma )$, where $\Sigma$ is specified below in (13).

In this work, we focus on the GHL test with $G=10$ groups. However, the impact of the choice of the number of groups is a well-known limitation of group-based test statistics. In the long history of the HL test, most sources use $G=10$, and in our limited investigation we found no evidence that this is a bad choice. As informal guidance, we remark that Bilder and Loughin (2014) suggest trying a few different values of *G* to make sure that a single result is not overly influenced by unfortunate grouping.

### GHL test statistic limiting distribution

We first state conditions used in our main theorem on the limiting distribution of $X^2_\text {GHL}$ under the null hypothesis (4). The theorem describes sufficient conditions for the convergence in distribution given in (12). Conditions (A), (C), and the first inequality in (B) below come from Stute and Zhu (2002). We assume the conditional density of *Y* given *X* is given by (2) or (3), with respect to some dominating measure, say $\nu$. For models without a dispersion parameter, the score function from observation *i* is $U_i(\beta _0)$, given by $U_i(\beta _0) = X_i m'(\beta _0^\top X_i)\left\{ \left( Y_i - m(\beta _0^\top X_i\right) \right) /v\left( m(\beta _0^\top X)\right) \}$, and the Fisher information matrix is $I_1(\beta _0) = {{\,\textrm{E}\,}}\left[ X X^\top \{m'(\beta _0^\top X)\}^2 /v\left( m(\beta _0^\top X)\right) \right]$.

**Condition (A)**

1. $I_1(\beta _0)$ exists and is positive definite.
2. Let $\ell (X_i,Y_i,\beta _0) = [I_1(\beta _0)]^{-1} U_i(\beta _0)$. Under the null hypothesis, we have
  $$\begin{aligned} n^{1/2} \{\beta _n - \beta _0\} = n^{-1/2} \sum _{i=1}^n \ell (X_i,Y_i, \beta _0) + o_P(1). \end{aligned}$$

**Condition (B)**: The function *m* is twice continuously differentiable and the function *v* is continuously differentiable. For some $\delta >0$ we have

$$\begin{aligned} 1)\quad {{\,\textrm{E}\,}}&\left[ \sup \left\{ \max _j\left|X_j\ m'(\beta ^\top X)\right|:\Vert \beta -\beta _0\Vert \le \delta \right\} \right]< \infty . \\ 2)\quad {{\,\textrm{E}\,}}&\left[ v^2(m(\beta _0^\top X))\right]< \infty , \\ 3)\quad {{\,\textrm{E}\,}}&\left[ X^\top X\sup \left\{ w_A^2(\beta ^\top X): \Vert \beta -\beta _0\Vert \le \delta \right\} \right]< \infty , \\ 4)\quad {{\,\textrm{E}\,}}&\left[ X^\top X \left\{ m'(\beta _0^\top X) \right\} ^2\right]< \infty . \\ 5)\quad {{\,\textrm{E}\,}}&\left[ \left( X^\top X\right) ^2 \sup \{ w_B^2(\beta ^\top X)): \Vert \beta -\beta _0\Vert \le \delta \}\right]< \infty , \text { and} \\ 6)\quad {{\,\textrm{E}\,}}&\left[ X^\top X\sup \left\{ |w_C(\beta ^\top X)|: \Vert \beta -\beta _0\Vert \le \delta \right\} \right] < \infty , \end{aligned}$$

where

$$\begin{aligned} w_A(u) = m'(u) v'(m(u)), \quad w_B(u)=m''(u), \quad \text {and} \quad w_C(u) = \frac{d}{du} \frac{\{m'(u)\}^2}{v(m(u))}. \end{aligned}$$

**Condition (C)**: Define ${\tilde{H}}(u, \beta ) ={{\,\textrm{E}\,}}\left\{ \sigma ^2(X) \mathbbm {1}(\beta ^\top X \le u)\right\}$. Then, ${\tilde{H}}$ is uniformly continuous in *u* at $\beta _0$ (and $\phi _0$). This condition requires that $\beta _0^\top X$ have a continuous distribution; in particular, $\beta _0 \ne 0$.

**Condition (D)**: Under the null hypothesis, with $\Sigma$ as defined below,

1. $$\Sigma _n \xrightarrow {p} \Sigma $$, and
2. $$\text {rank}(\Sigma _n) \xrightarrow {p} \text {rank}(\Sigma )$$.

We specify $\Sigma$ when $f_{Y\mid X}$ is as in (2) or (3). Define the column vector

$$\begin{aligned} q(x, \beta ) = \frac{\partial m(\beta ^\top x)}{\partial \beta } = (q_1(x,\beta ), \ldots , q_d(x,\beta ))^\top =m'(\beta ^\top x) x. \end{aligned}$$

Define the vector-valued function $Q^\top = (Q_1, \ldots , Q_d)$ by

$$\begin{aligned} Q_i(u) \equiv Q_i(u, \beta _0) = {{\,\textrm{E}\,}}(q_i(X, \beta _0) \mathbbm {1}(\beta _0^\top X \le u)), \quad 1 \le i \le d. \end{aligned}$$

The matrix $\Sigma$ is defined by

13 $$\begin{aligned} \Sigma = \Sigma ^{(1)} - \Sigma ^{(2)}, \end{aligned}$$

where, for $$1 \le g, g' \le G$$,

$$\begin{aligned} \Sigma _{gg'}^{(1)}&= {\left\{ \begin{array}{ll} {{\,\textrm{E}\,}}\left[ v(m(\beta _0^\top X))\mathbbm {1}(k_{g-1} < \beta _n^\top X \le k_g)\right] , &{} g = g' \\ 0, &{} g \ne g' \end{array}\right. }\\ \Sigma _{gg'}^{(2)}&= (Q(k_g) - Q(k_{g-1}))^\top [I_1(\beta _0)]^{-1} (Q(k_{g'}) - Q(k_{g'-1})). \end{aligned}$$

In the supplementary material, we prove our main theorem.

#### Theorem 1

Suppose that ${{\,\textrm{E}\,}}(Y^2) < \infty$, conditions (A), (B), and (C) hold, and that $\phi _n \xrightarrow {p} \phi _0$. Assume the cell boundaries $k_{n,g}$ satisfy $k_{n,g} \xrightarrow {p} k_g$ for $g=0,\ldots ,G$ and that the $k_g$ are distinct. Then, under the null hypothesis given by (4), with $f_{Y\mid X}$ as in (2) or (3), we have

$$\begin{aligned} S_n^1 \xrightarrow {d} \text {MVN}_G(0, \phi _0\Sigma ) \equiv S_\infty ^1, \end{aligned}$$

where $S_n^1$ is as defined in (7), and $\Sigma$ is given by (13).

If there exists any sequence of matrices $\Sigma _n$ that satisfies condition (D), then, putting $r = {{\,\textrm{rank}\,}}(\Sigma )$,

$$\begin{aligned} S_n^{1 \top } \Sigma _n^+ S_n^1 /\phi _n\xrightarrow {d} \chi _r^2.\ \end{aligned}$$

If conditions (A), (B), and (C) hold, then the particular matrix $\Sigma _n$ in (10) converges almost surely to $\Sigma$, so (D1) holds under the null hypothesis. Finally, if conditions (i) and (ii) of Sect. 3.4 are satisfied, then conditions (B) and (C) hold.

### GLMs for which the GHL test is valid

Formally, conditions (A), (B), (C), and (D) should be verified before using the generalized HL test statistic, $X^2_{\text {GHL}}$. From Theorem 1, the conditions below are *sufficient* for the validity of conditions (B) and (C) provided that $f_{Y\mid X}$ is of the form presented in (2) or (3):

(i) One of the distribution/link function combinations from Table 1 is used.
(ii) The joint probability distribution of the explanatory variables, *X*, has compact support. For all $\beta$ in an open neighbourhood $\mathcal{N}$ of $\beta _0$ the variable $\beta ^\top X$ has a bounded continuous Lebesgue density, the support, $\textrm{supp}(\beta ^\top X)$, of the linear predictor is an interval, and $P(m(\beta ^\top X )\in \Theta )= 1$.

The supplementary material outlines how to verify conditions (A), (B), (C), and (D), weaken the compactness assumption in (ii), and extend our test to other GLMs.

**Table 1:** Several possible distribution and link function combinations. Distributions are parameterized so that $\mu$ or $\pi$ represents the mean of the distribution. *Gamma distribution with variance $\mu ^2/k$. **Inverse Gaussian distribution with variance $\mu ^3/\lambda$. ***Negative binomial distribution with variance $\mu + \mu ^2/k$. For the negative binomial distribution, *k* is assumed to be known

Distribution	Example possible links
Normal$(\mu ,\sigma ^2)$	identity
Bernoulli$(\pi )$	logit, probit, cauchit, cloglog
Poisson$(\lambda )$	log, square root
Gamma($\mu ,k$)*	log
IG($\mu ,\lambda$)**	log
NB($\mu ,k$)***	log

### Consistency of the GHL test

We discuss power in terms of consistency; we outline one possible set of conditions on the alternative distribution, the specific model, and the choice of cell boundaries that will ensure that our test is consistent. Precise versions of conditions (K1-5) that follow are in the supplementary material.

Our conclusions are affected by the presence or absence of an intercept term. Here, we present results for models that do not have an intercept. For such models, we assume that the rows $X_i$ of the design matrix are i.i.d. and have a Lebesgue density. Models with intercepts are discussed in the supplementary material. We let $\eta (x) = \beta ^\top x$ denote the linear predictor of our GLM.

#### Behaviour of coefficient estimator under the alternative

In the supplementary material, we give conditions (K1), used to check conditions in White (1982), which guarantee that the estimate $\beta _{n}$ has a limit under the alternative being considered; we denote this limit by $\beta ^*$. The most important additional components are a restriction to a compact parameter set not containing $\beta =0$, say ${\textbf{B}}$, uniformity over ${\textbf{B}}$ in some of our conditions, and the assumption that *X* has a density. From White (1982), conditions (K1) are enough to ensure the existence of a (possibly not unique) maximizer of the GLM likelihood.

Also in the supplementary material, we give conditions (K2) on the joint density of *X* and *Y* which come, essentially, from White (1982). The two conditions (K1) and (K2) now imply Assumptions A1, A2, and A3 of White (1982). In turn, these imply almost sure convergence of $\beta _n$ to a unique $\beta ^* \in {\textbf{B}}$. We write $\eta ^*(X)$ for the linear predictor evaluated at $\beta ^*$. That is, $\eta ^*(X) = \beta ^{*\top } X$.

#### Behaviour of interval endpoints under the alternative

We consider both fixed and random interval endpoints. Our consistency results need assumptions about the probability that $\eta (X)$ belongs to each limiting interval; these probabilities depend on $\beta$ and the assumptions will be false for $\beta =0$. For some choices of intervals, there can be intervals that have no observations almost surely. We need to assume that this does not happen for the predictor $\eta ^*$. In the supplementary material, we present condition (K3), which strengthens our main condition (C); this condition requires $\beta ^* \ne 0$.

There may be $\beta \in {\textbf{B}}$ such that $\textrm{supp}(\eta )$ is bounded; in that case some methods of choosing boundaries (like fixed boundaries) will not satisfy (K3). However, the method used in our simulations chooses cell boundaries using the estimate $\beta _n$ so as to make all the cells have approximately the same sum of variances of the responses. For this method, (K3) holds.

#### Behaviour of covariance estimate under the alternative

We consider our estimate $\Sigma _n$ of $\Sigma$. Let $\Sigma _n(\beta )$ and $\Sigma (\beta )$ be the matrices in (10) and (13) evaluated at a general $\beta$; as before, $\Sigma _n = \Sigma _n(\beta _n)$. Condition (B) imposes moment conditions in a neighbourhood implying our estimate $\Sigma _n$ is consistent for $\Sigma (\beta _0)$. Assumption (K4) extends these conditions to every $\beta \in {\textbf{B}}$ so that they apply to the unknown value $\beta ^*$. Under conditions (K1–4), our arguments show that, with probability equal to 1, $\Sigma _n(\beta ) \rightarrow \Sigma (\beta )$ uniformly in $\beta \in {\textbf{B}}$; moreover, $\Sigma$ is a continuous function of $\beta$ so $\Sigma _n$ converges to $\Sigma (\beta ^*)$.

In the supplementary material, we show that under reasonable conditions the matrix $\Sigma (\beta )$ has rank *G* or $G-1$ and that when *n* is large $\Sigma _n(\beta )$ has the same rank with high probability. We delineate the special cases where rank $G-1$ arises. Here we give one common special case of our results; a more general version is in the supplementary material.

##### Theorem 2

Fix $\beta \in {\textbf{B}}$. Assume conditions (K1), (K3), and (K4) and that we are not fitting an intercept. Then, $\textrm{rank}(\Sigma (\beta )) = G$ unless there is a constant *c* such that

14 $$\begin{aligned} v(m(u)) = m'(u) cu \end{aligned}$$

for all $u\in \textrm{supp}(\eta (X))$. If (14) holds for all $u \in \textrm{supp}(\eta (X))$, then $\textrm{rank}(\Sigma (\beta )) = G-1$.

We turn to the rank of $\Sigma _n(\beta )$. Unless identity (14) holds on $\textrm{supp}(\eta (X))$, the rank of $\Sigma (\beta )$ is *G* under our conditions. Since $\Sigma _n$ converges to $\Sigma (\beta ^*)$ and $\beta ^*\ne 0$ is in ${\textbf{B}}$, we find that $P(\textrm{rank}(\Sigma _n(\beta )) = G)$ converges to 1 in this case.

#### Our consistency theorem

Consistency requires that we have modelled the mean incorrectly in a fairly strong sense. For $1\le g \le G$, define

$$\begin{aligned} \delta _g(\beta ) = {{\,\textrm{E}\,}}{\left\{ \mathbbm {1}(k_{g-1} < \eta (X) \le k_g) \left\{ Y-m(\eta ( X))\right\} \right\} }. \end{aligned}$$

Also define $$ {\bar{\delta }}(\beta ) = \frac{1}{G} \sum _{g=1}^G \delta _g(\beta )$$.

**Condition (K5)**: The model fitted does not have an intercept and either:

1. For every *c* the set $\{u\in \textrm{supp}(\beta ^{*\top } X)\}$ where identity (14) does not hold has positive Lebesgue measure. For all $\beta \in {\textbf{B}}$, $\sum _g \left( \delta _g(\beta )\right) ^2 > 0$; or
2. Identity (14) holds for all *u*, and $\sum _g \left( \delta _g(\beta ) -{{\bar{\delta }}}(\beta )\right) ^2> 0$ for all $\beta \in {\textbf{B}}$.

##### Theorem 3

Under conditions (K1), (K2), (K3), (K4), and (K5),

$$\begin{aligned} X^2_{\text {GHL}} \xrightarrow {p} \infty . \end{aligned}$$

We note that in the above theorem, the test based on $X^2_{\text {GHL}}$ is consistent against the alternative in question. That is, for any $\alpha \in (0, 1)$, under $H_1$ we have $P(X^2_{\text {GHL}} > \chi ^2_{r, 1-\alpha }) \rightarrow 1$ as $n \rightarrow \infty$, where $r = \text {rank}(\Sigma )$.

## Simulation study design

We perform a simulation study to assess the performance of our proposed test. The purpose of this paper is to extend the HL test to a wide array of new distribution models. We therefore focus on applications to data generated from Poisson distributions, since Poisson appears to be a common non-binomial GLM, but we include other response distributions such as the gamma and inverse Gaussian distributions. While this paper does not study the performance of the GHL test in binomial models, we do compare the performance of the HL test and the binomial version of the GHL test in Surjanovic and Loughin (2021). That work arose because we discovered that certain plausible data structures cause the regular HL test to fail, whereas the binomial version of the GHL test appropriately resolves the issue. We carefully document that problem and discuss it in Surjanovic and Loughin (2021) as a warning to practitioners who routinely apply HL to binomial models.

The binomial version of the GHL test is also studied in Hosmer and Hjort (2002), where it is equivalent to their $X^2_w$ test when all weights are set to 1. Full details of simulation settings are in the following sections and the supplementary material. We first give an overview and describe features common to all experiments.

Unless otherwise specified, the mean is taken to be $m(\beta ^T x)$ where $m^{-1}$ is the log link, and the null hypothesis is that the Poisson distribution with this structure is correct. We compare rejection rates under different null and alternative hypothesis settings for four different GOF tests: the naive generalized HL test, our new generalized HL test (GHL), the Stute-Zhu (SZ) test, and the Su-Wei (SW) test. For HL and GHL, we use $G=10$ unless specified otherwise. The naive generalized HL test is included to demonstrate that a test without proper theoretical justification may fail. We believe that the SZ test has an appealing construction, but is perhaps not as well known as the SW test, which can be found in other simulation studies. These two tests are included because they do not rely heavily on kernel-based density estimation and are relatively straightforward to implement.

For $x,v \in {\mathbb {R}}^d$ let $\mathbbm {1}(x\le v)$ be 1 if and only if $x_j \le v_j$ for all $j=1,2,\ldots ,d$. Define

$$\begin{aligned} \widetilde{R_n}(v) = \frac{1}{\sqrt{n}} \sum _{i=1}^n \mathbbm {1}(X_i \le v) [Y_i - m(\beta _n^\top X_i)]. \end{aligned}$$

Using our notation, the SW test statistic is defined as

15 $$\begin{aligned} X^2_{\text {SW}} = \sup _{v \in {\mathbb {R}}^d} |\widetilde{R_n}(v) |. \end{aligned}$$

The SZ test statistic has a more complicated form as a Cramér–von Mises statistic applied to a specially transformed version of the $R_n^1$ process. We also slightly modify the SZ test statistic to detect overdispersion in the Poisson case. These test statistics, including the modification to SZ, are described in greater detail in the supplementary material.

We use sample sizes of 100 and 500 throughout this simulation study, representing moderate and large sample sizes in many studies in medical and other disciplines where GLMs are used. Unless otherwise stated, only the results for the sample size of 100 are reported for the null and power simulation settings; important differences between the two settings are summarized in Sect. 5. For each simulation setting, we produce 2500 realizations. On each realization, we record a binary value for each test indicating whether the test rejects the null hypothesis for that data set. The proportion of the 2500 realizations for which a test rejects the null hypothesis estimates the test’s true probability of type I error or power in that setting. In the null simulations, approximate 95% confidence intervals for the binomial probability of rejection $H_0$ can be obtained from the observed rejection rates by adding and subtracting 0.009 ($1.96 \times \sqrt{0.05 \cdot 0.95/2500} \approx 0.009$). Conservative 95% confidence intervals for power can be obtained from the observed rejection rates by adding and subtracting 0.02, accounting for the widest interval, when a proportion is equal to 0.5 ($1.96 \times \sqrt{0.5 \cdot 0.5/2500} \approx 0.02$). The simulations are performed using R.

### Null distribution

Under the null hypothesis described by (4), where $f_{Y \mid X}$ is a Poisson distribution, we consider six settings with a log link and three with a square root link, varying the distribution of explanatory variables and the true parameter values in each case. In the first three null settings with a log link, a model with a single covariate, *X*, and an intercept term is used. These settings serve to examine the effect of small and large fitted values on the null distribution of the test statistics. The distribution of *X* and values of $\beta _0$ and $\beta _1$ are chosen so that the fitted values take on a wide range of values (approximately 0.1 to 100) in the first setting, are moderate in size (approximately 1 to 10) for the second setting, and are very small (approximately 0.1 to 1) for the third setting. Specifics on these and other settings are given in the supplementary material.

For settings 4, 5, and 6, coefficients are chosen so that the fitted values are moderate in size (rarely less than 1, with an average of approximately 4 or 5), so that other sources of potential problems for the GOF tests can be explored. The fourth setting examines a model including two continuous covariates and one dichotomous covariate (the “Normal-Bernoulli” model), and is similar to the one used in Hosmer and Hjort (2002). The fifth and sixth simulation settings examine the effects of correlated and right-skewed covariates, respectively. It is well known that in the presence of multicollinearity the variance of regression parameter estimates can become inflated. The correlated covariates setting is included to assess the impact of multicollinearity on the GOF tests, since the estimated covariance matrix of the regression coefficients is used in the calculation of the GHL test statistic. The right-skewed covariate in setting 6 is included in order to assess its potential impact on the SZ test, since this test makes use of kernel-based density estimation as a part of the calculation of the test statistic, albeit in a one-dimensional case.

We next create three settings labelled 1b, 2b, and 3b, that are the same as settings 1, 2, and 3, respectively, except the true and fitted models use a square root link, rather than a log link. We also use simulations to verify that the proposed GHL test maintains its size in models where there are unknown dispersion parameters that must be estimated. We consider several settings with gamma, inverse Gaussian, and negative binomial responses with a single covariate, log link, and moderate-sized means. We fix the true dispersion parameter to be $\phi _0 = 0.1$, so that the variance of the response distribution given its mean, $\mu$, is $\phi _0 \mu ^2$, $\phi _0 \mu ^3$, and $\mu + \phi _0 \mu ^2$, for the gamma, inverse Gaussian, and negative binomial responses, respectively. The dispersion parameter for the gamma and inverse Gaussian models is estimated using a weighted average of the squared residuals, which is the default in summary.glm(). The negative binomial distribution with an *unknown* dispersion parameter is a popular alternative to Poisson regression that does not fall within the exponential dispersion family framework presented. In this case, the parameter that controls the variance is estimated using maximum likelihood, which is the default estimation procedure in MASS::glm.nb() in R.

Finally, we consider simulation settings that examine the performance of the GHL and naive generalized HL tests when only discrete covariates are present. We repeat setting 2 above, except that *X* is sampled uniformly from 30 or 50 possible values on a grid. These numbers of points are chosen so that the data can be split into at least $G=10$ groups. All model coefficients and further details of the simulation study, such as the implementation of the GHL test for the negative binomial model, are given in the supplementary material.

### Power

To examine the power of the GOF tests, we consider four types of deviations from the null model: a missing quadratic term, overdispersion, a missing interaction term, and an incorrectly specified link function. These settings are similar to those used in Hosmer and Hjort (2002) and are realistic model misspecifications. In the first three settings, the severity of deviation between the true model and the fitted model is controlled by regression parameters to represent four levels ranging from “small” to “large” deviations from the assumed additive linear model. In the incorrect link setting, the true model uses a square root link, but the fitted model assumes a log link. In all four settings, we use a Poisson GLM and choose appropriate regression coefficients so that the fitted values are moderate in size, rarely less than 1 and often smaller than 10, to ensure that a large rejection rate is not simply due to small fitted values in the Pearson-like test statistics. All four power simulation settings are described in detail in the supplementary material.

### Performance with larger models

We additionally assess the performance of each of the tests with larger models. The theoretical results presented in this work assume a constant dimension, *d*, and consider limiting distributions as the sample size tends to infinity. Here, we again consider fixed *d*, but as a factor worth studying on its own.

Realizations of *Y* are again drawn from a Poisson distribution with a log link and $d=2,10,20,30,40,50$ parameters. We use sample sizes $n=100$ and 500. To keep the distribution of the fitted values approximately constant as *d* is varied, we set $\beta _0 = 1.67$, and $\beta _1 = \ldots = \beta _{d-1} = \sqrt{0.0717/(d-1)}$. This gives a distribution of fitted values mostly within the interval [1, 10], ensuring that expected counts within each group used in the calculation of the Pearson statistic are sufficiently large. The SW test is omitted due to computational challenges that arise with this test with large models and because we observe that a large proportion of the data needs to be omitted when *d* is large and $n=100$ for the test statistic to be computed (see the supplementary material).

## Simulation results

### Null distribution

From the null simulation results in Table 2, we see that the estimated type I error rate for the GHL test does not significantly differ from the nominal level, since all values are in the interval (0.041, 0.059). However, the naive generalized HL test falls out of this interval in three settings, whereas the SW and SZ tests fall out of this interval in two and four settings, respectively. Interestingly, even with $d=4$ in setting 4, we begin to see a decreased type I error rate for the naive generalized HL test, a phenomenon discussed in more detail later in this section. However, with a sample size of 500, the naive generalized HL test and the SZ test generally have better empirical rejection rates, whereas the SW test has similar poor performance even with a larger sample size. Table 3 summarizes type I error rates for the GHL test in the presence of a dispersion parameter. A larger sample size is sometimes needed to ensure that the finite sampling distribution of the test statistic is well approximated by its limiting chi-squared distribution. In our simulation settings, the estimated type I error rate is closer to the nominal rate for the larger sample size of 500. Type I error rates for settings with discrete covariates are in Table 4. The naive generalized HL test and the GHL test hold their size for the considered sub-settings.

**Table 2:** Estimated type I error rates (null setting simulation results)

Statistic/setting	1	1b	2	2b	3	3b
${\widehat{C}}_G^*$	0.058	*0.061*	0.042	0.051	0.051	0.047
$X^2_{\text {GHL}}$	0.053	0.056	0.049	0.052	0.052	0.050
$X^2_{\text {SW}}$	0.043	*0.062*	*0.041*	0.052	0.042	0.048
$X^2_{\text {SZ}}$	*0.032*	0.045	*0.039*	*0.039*	0.044	*0.041*

Statistic/setting	4	5	6			
${\widehat{C}}_G^*$	*0.037*	0.055	*0.059*			
$X^2_{\text {GHL}}$	0.048	0.056	0.054			
$X^2_{\text {SW}}$	0.051	0.048	0.053			
$X^2_{\text {SZ}}$	–	0.045	0.049			

Numbers in italics represent cases where the estimated rejection rate is significantly different from 0.05 (i.e., outside of the interval (0.041, 0.059))

**Table 3:** Estimated type I error rates for the GHL test in the presence of a dispersion parameter for samples of size $n=100$ and $n=500$. For the negative binomial response with $n=100$, approximately 3% of simulation draws were discarded due to GLM convergence warnings

Distribution	$n = 100$	$n = 500$
Gamma	0.041	0.042
Inverse Gaussian	*0.064*	0.055
Negative binomial	0.042	0.053

**Table 4:** Estimated type I error rates for the naive generalized HL and GHL tests when all covariates are discrete. Sample sizes $n=100$ and $n=500$ are considered, with the discrete covariate being a random sample of size *n* from a uniformly spaced sequence of length $n_\text {points}$ on $[-3,3]$

Statistic	$n = 100$	$n = 500$
${\hat{C}}_G^*$ ($n_\text {points} = 30$)	0.051	0.051
${\hat{C}}_G^*$ ($n_\text {points} = 50$)	0.053	0.042
$X^2_\text {GHL}$ ($n_\text {points} = 30$)	0.052	0.052
$X^2_\text {GHL}$ ($n_\text {points} = 50$)	0.050	0.044

### Power

**Fig. 1:**
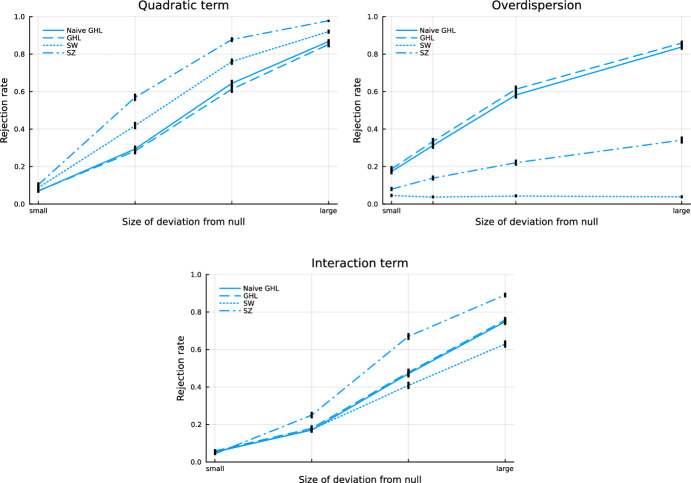
Power simulation results for the first three settings. Solid red lines are 95% Wilson CIs for the mean rejection rate (colour figure online)

The power simulation results displayed in Fig. 1 show our new test does have power to detect each of the violated model assumptions we tested. However, for those model flaws that are detectable by the SZ and SW tests, these two tests generally have better power than the tests based on grouped residuals. In the simulation settings we explored, our test had power to detect overdispersion while the SW and SZ tests had little or no power, although these two competitors were not necessarily designed to detect overdispersion.

### Larger models

The null distribution of the naive generalized HL test statistic, ${\widehat{C}}_G^*$, is not well approximated by the usual $\chi ^2_{G-2}$ distribution in this setting with a finite sample size. The impact of the number of parameters on the estimated mean and level of the naive generalized HL test statistic can be seen in Table 5, where the $G-2$ degrees of freedom approximation for this test deteriorates as the model size grows, relative to the sample size. This adverse effect is less pronounced with a larger sample size. The estimated type I error rates steadily decrease for the naive generalized HL test from about 0.050 to 0.001 as *d* grows from 2 to 50 with a sample size of 100, and down to 0.030 with a sample size of 500. Our proposed test does not seem to be affected by the number of parameters present in the model. Similar results were obtained for both tests with $G=50$, used to ensure that $G>d$, as is required by the traditional HL test on which the naive GHL test is based.

**Table 5:** Estimated means (top) and levels (bottom) of the naive HL and generalized HL statistics for Poisson regression models with $n \in \{100, 500\}$ and $G=10$

Statistic	*n*	$d=2$	$d=10$	$d=20$	$d=30$	$d=40$	$d=50$
${\widehat{C}}_G^*$	100	8.05	7.24	6.48	5.61	4.76	3.85
$X^2_{\text {GHL}}$	100	8.99	8.89	9.03	9.09	9.20	9.13
${\widehat{C}}_G^*$	500	8.02	7.86	7.81	7.57	7.34	7.23
$X^2_{\text {GHL}}$	500	8.98	8.95	9.09	9.09	9.02	9.02

Statistic	*n*	$d=2$	$d=10$	$d=20$	$d=30$	$d=40$	$d=50$
${\widehat{C}}_G^*$	100	0.050	0.030	0.012	0.005	0.002	0.001
$X^2_{\text {GHL}}$	100	0.044	0.049	0.048	0.053	0.054	0.056
${\widehat{C}}_G^*$	500	0.049	0.047	0.045	0.036	0.030	0.030
$X^2_{\text {GHL}}$	500	0.050	0.043	0.056	0.054	0.051	0.054

For the naive HL and generalized HL test statistics, the means should be approximately $G-2 = 8$ and $G-1 = 9$, respectively. In all cases, the type I error rate should be $\alpha = 0.05$

## Discussion

The simulation results of Sect. 5 show that the GHL test provides competitive or comparable power in various simulation settings. Our test is also computationally efficient, straightforward to implement, and works in a variety of scenarios. There is no need for a choice of a kernel bandwidth (although the choice of number of groups, *G*, can play a role in determining the outcome of the test), and the output can be interpreted in a meaningful way by assessing differences between observed and expected counts in each of the groups. The naive generalization of the HL test does not work well under certain settings, but use of the GHL test resolves these issues.

### Supplementary Information

Below is the link to the electronic supplementary material.

Supplementary file 1 (pdf 368 KB)
