# Supplementary material for: A generalized Hosmer–Lemeshow goodness-of-fit test for a family of generalized linear models
Source: Test (Madr). 2023 Dec 19;33(2):589–608. doi: 10.1007/s11749-023-00912-8 (PMC11164741; doi:10.1007/s11749-023-00912-8)
Supplement: Supplementary file 1 — (pdf 368 KB) [file 11749_2023_912_MOESM1_ESM.pdf]

Supplementary Material: A Generalized Hosmer-Lemeshow Goodness-of-Fit Test for a Family of Generalized Linear Models

Nikola Surjanovic[1]*, Richard A. Lockhart[2], and Thomas M. Loughin[2]*****

[1]Department of Statistics, University of British Columbia, Vancouver, British Columbia, Canada
[2]Department of Statistics and Actuarial Science, Simon Fraser University, Burnaby, British Columbia, Canada
**email:* nikola.surjanovic@stat.ubc.ca
***email:* lockhart@sfu.ca
****email:* tloughin@sfu.ca

Contents

S.1 Supplementary Material Appendix A: Additional Results and Implementation Details 2

S.1.1 Power Simulation Settings . . . 2
S.1.2 Further Details . . . 3
S.1.3 Assorted Tables . . . 4
S.1.4 Description of GOF Test Competitors . . . 4
S.1.5 Application . . . 7

S.2 Supplementary Material Appendix B: Discussion, Extensions, and Proofs 9

S.2.1 Proof of Theorem 1 . . . 9
S.2.1.1 Restatement of Theorem 1 . . . 9
S.2.1.2 Proof of Statement 1 . . . 11
S.2.1.3 Proof of Statement 2 . . . 12
S.2.1.4 Proof of Statement 3 . . . 13
S.2.1.5 Proof of Statement 4 . . . 18
S.2.2 Verifying Conditions (A), (B), and (C) for Various GLMs . . . 18
S.2.2.1 Condition (A) . . . 19
S.2.2.2 Condition (B) . . . 19
S.2.2.3 Condition (C) . . . 26
S.2.3 Condition (D) and the rank of the covariance . . . 26
S.2.3.1 Condition (D2) . . . 27
S.2.3.2 Rank of the covariance matrix . . . 27
S.2.3.3 Rank of the covariance estimator . . . 31

S.2.4 Consistency of our test 34
S.2.4.1 Behaviour of coefficient estimator under the alternative 34
S.2.4.2 Behaviour of interval endpoints under the alternative 36
S.2.4.3 Behaviour of covariance estimator under the alternative 36
S.2.4.4 Consistency 37
S.2.5 Empirical Process Lemmas 40

Note: Equations, theorems, sections, etc. in the supplementary material are numbered (S.1), (S.2), ... and equations without an "S" refer to the main text.

S.1 Supplementary Material Appendix A: Additional Results and Implementation Details

S.1.1 Power Simulation Settings

In this section we describe the four power simulation settings in greater detail. For each setting, we use J to represent the sub-settings, where larger values of J represent greater deviations from the null hypothesis, subjectively labeled from "small" to "large" within each setting.

For the missing quadratic term in setting 1, the true model is

$$\mathrm{E}(Y|X=x) = \exp(\beta_0 + \beta_1 x + \beta_2 x^2),$$

but we omit the quadratic term when fitting the model. In this setting, we use $X \sim U(-3, 3)$. Four sub-settings are considered, in order to investigate the impact of varying degrees of non-linearity in the linear predictor. The coefficients $\beta_0, \beta_1, \beta_2$ are chosen so that the mean of Y is J, 5, and 8 when $X = -3, 0$ and -3 , respectively. We use $J = 4, 6, 8, 10$, representing increasing non-linearity in the linear predictor.

In order to simulate overdispersion for setting 2, we draw realizations of Y given X from a negative binomial distribution with mean

$$\mathrm{E}(Y|X=x) = \exp(\beta_0 + \beta_1 x),$$

and variance

$$\mathrm{Var}(Y|X=x) = \mathrm{E}(Y|X=x) + J\,\mathrm{E}(Y|X=x)^2$$

for $J = 1/16, 1/8, 1/4, 1/2$. The fitted model is Poisson with the same mean structure as the negative binomial. Larger values of J represent greater overdispersion. In this setting we also use $X \sim U(-3, 3)$.

For setting 3, the true model is

$$\mathrm{E}(Y|X=x, B=b) = \exp(\beta_0 + \beta_1 x + \beta_2 b + \beta_3 x b),$$

with $X \sim U(-3,3)$ and $B \sim$ Bernoulli(0.5). The fitted model excludes the final interaction term. Four sub-settings are considered for setting 3, similar to setting 1. The coefficients are chosen so that the mean of Y is equal to 5, 5, 7, and J when $(X, B) = (-3,0), (-3,1), (3,0)$, and $(3,1)$, respectively. We use $J = 8, 12, 16, 20$, representing increasing amounts of interactivity.

Finally, in order to assess the effect of an incorrectly specified link function in setting 4, we consider the square root and identity link functions as two sub-settings, fitting a log-linear Poisson model for both. We have $X \sim U(-3,3)$, and the coefficients are chosen so that the conditional mean of Y is 5 and 8, when $X = 0$ and 3, respectively.

S.1.2 Further Details

Implementation of the Interval Endpoint Selection Method

The HL test statistic can be viewed as a sum of Pearson residuals where the denominator in each term includes an estimate related to the average response variance in a given group. To prevent instabilities in the test statistic when the average variance is small, a special interval endpoint selection method is used in order to keep $\sum_{i=1}^{n} \hat{\sigma}^2(x_i) I_i^{(g)}$ roughly constant across groups. The same interval endpoint selection procedure is used for the GHL test. The implementation is based on the "weighted.quantile()" function from the "spatstat" package in R. Groups with no observations can occur, for example, when a very large fitted value is present. To prevent this, the weighted $(G-1)/G \times 100$th percentile is obtained first. Then, observations that fall into this group are removed, and the weighted $(G-2)/(G-1) \times 100$th percentile is obtained from the remaining data. This process is repeated until G groups are formed.

GLM Convergence with Non-canonical Links

When fitting a Poisson GLM with identity or square-root link in R, certain issues can arise. In general, we aid convergence of the parameter estimates for these non-canonical link models by providing starting values such as rounded versions of the true parameter values to the "glm()" function call. On occasion, fitting the GLM with a noncanonical link results in a warning, in which case the particular simulation realization is omitted, resulting in fewer than 2500 simulation realizations. For example, warnings such as "step size truncated: out of bounds" and "glm.fit: algorithm stopped at boundary value" occurred, among other warnings. In addition, for the null simulations with a dispersion parameter, warnings occurred for the setting with a negative binomial response and a sample size of $n = 100$ where less than 3% of the simulation realizations were discarded.

GHL Test Statistic for Negative Binomial Responses

The negative binomial distribution with an *unknown* dispersion parameter does not fall into the exponential

dispersion family framework presented in the paper. However, in the simulation study we include such a setting to examine the performance of the GHL test. In this case, the test statistic remains the same except that we redefine the matrices

$$V_n^{1/2} = \operatorname{diag}\left(\left[m(\beta^\top x_i) + \phi \cdot m(\beta^\top x_i)^2\right]^{1/2}\right)\Big|_{(\beta,\phi)=(\beta_n,\phi_n)},$$
$$W_n^{1/2} = \operatorname{diag}\left(\frac{m'(\beta^\top x_i)}{\left[m(\beta^\top x_i) + \phi \cdot m(\beta^\top x_i)^2\right]^{1/2}}\right)\Bigg|_{(\beta,\phi)=(\beta_n,\phi_n)},$$

because the conditional variance of the response can no longer be written in the form $\phi \cdot v(m(\beta^\top x))$. The test statistic is obtained from (11) without the division by ϕ_n.

Normal-Bernoulli model

The Normal-Bernoulli model includes two continuous covariates, X_1 and X_2, along with a dichotomous covariate, D. Here we have $X_i \sim N(\mu_i, \Sigma)$, where $\mu_1 = (-1, -1)$, $\mu_2 = (1, 1)$, and $\Sigma = (1, 0.5; 0.5, 1)$. The dichotomous covariate is $D \sim \text{Bernoulli}(0.5)$. The true linear predictor is $\beta_0 + \beta_1 X_1 + \beta_2 X_2 + \beta_3 D$, where the parameter values are described in the tables below.

Correlated covariates

The true linear predictor for this model is $\beta_0 + \beta_1 X_1 + \beta_2 X_2$, where (X_1, X_2) are drawn from a multivariate normal distribution with mean $(0, 0)$ and marginal variances equal to one. The correlation between X_1 and X_2 is set to be $\rho = 0.7$.

S.1.3 Assorted Tables

Supplementary tables S.1, S.2, S.3, S.4, and S.5 are included below.

Supplementary Table S.1: Link and inverse link functions considered

Link function name	Link function form	Inverse link function form
identity	μ	$\beta^\top x$
log	$\log(\mu)$	$\exp(\beta^\top x)$
logit	$\text{logit}(\mu) = \log(\mu/(1-\mu))$	$\exp(\beta^\top x)/(1 + \exp(\beta^\top x))$
probit	$\text{probit}(\mu) = \Phi^{-1}(\mu)$	$\Phi(\beta^\top x)$
cauchit	$\text{cauchit}(\mu) = \tan(\pi(\mu - 1/2))$	$1/\pi \arctan(\beta^\top x) + 1/2$
cloglog	$\text{cloglog}(\mu) = \log(-\log(1-\mu))$	$1 - \exp(-\exp(\beta^\top x))$
square root	$\sqrt{\mu}$	$(\beta^\top x)^2$

S.1.4 Description of GOF Test Competitors

For the simulation study described in Section 4 of the paper, we compare our test to tests given by Su and Wei (1991) and Stute and Zhu (2002).

Supplementary Table S.2: Several conditional distributions of Y given X that can be written in the form of (2). *Negative binomial distribution with the dispersion parameter k assumed to be known.

Distribution	θ	Θ	$b(\theta)$	$v(m)$
Normal(μ, σ^2)	μ/σ^2	$(-\infty, \infty)$	$\theta^2\sigma^2/2$	1
Bernoulli(π)	$\log(\pi/(1-\pi))$	$(-\infty, \infty)$	$\log(1+e^\theta)$	$m(1-m)$
Poisson(λ)	$\log(\lambda)$	$(-\infty, \infty)$	e^θ	m
Gamma(μ, k)	$-k/\mu$	$(-\infty, 0)$	$-k\log(-\theta)$	m^2
IG(μ, λ)	$-\lambda/(2\mu^2)$	$(-\infty, 0)$	$-\sqrt{-2\theta\lambda}$	m^3
NB(μ, k)*	$\log(\mu/(\mu+k))$	$(-\infty, 0)$	$-k\log(1-e^\theta)$	$m+m^2/k$

Supplementary Table S.3: Null simulation settings

Setting	Distribution of Covariate(s)	True coefficients
1	$X \sim U(-3,3)$	$\beta_0 = 1.15, \beta_1 = 1.15$
1b	$X \sim U(-3,3)$	$\beta_0 = 5.16, \beta_1 = 1.61$
2	$X \sim U(-3,3)$	$\beta_0 = 1.15, \beta_1 = 0.384$
2b	$X \sim U(-3,3)$	$\beta_0 = 2.08, \beta_1 = 0.360$
3	$X \sim U(-3,3)$	$\beta_0 = -1.15, \beta_1 = 0.384$
3b	$X \sim U(-3,3)$	$\beta_0 = 0.658, \beta_1 = 0.114$
4	Normal-Bernoulli model	$\beta_0 = 1, \beta_1 = 0.2, \beta_2 = -0.2, \beta_3 = 0.7$
5	Correlated covariates	$\beta_0 = 1.70, \beta_1 = 0.148, \beta_2 = 0.148$
6	$X \sim \text{Exp}(1)$	$\beta_0 = 1.15, \beta_1 = 0.384$
Dispersion settings	$X \sim U(-3,3)$	$\beta_0 = 1.15, \beta_1 = 0.384$

Supplementary Table S.4: Power simulation settings

Setting	Description	True coefficients
1	Missing quadratic term	$\beta_0 = 1.61, \quad \beta_1 = 0.347 - 1/6\log(J),$<br>$\beta_2 = -0.0633 + 1/18\log(J)$
2	Overdispersion	$\beta_0 = 1.61, \beta_1 = 0.157$
3	Missing interaction term	$\beta_0 = 1.78, \quad \beta_1 = 0.0561, \quad \beta_2 = 1/2\log(J/5),$<br>$\beta_3 = 1/6\log(J/5)$
4	Incorrectly specified link	$\beta_0 = 2.24$, $\beta_1 = 0.197$ (square root)<br>$\beta_0 = 5$, $\beta_1 = 1$ (identity)

Supplementary Table S.5: Power simulation results - incorrect link function

Statistic / Link	Square root	Identity
$\widehat{C}_G^*$	0.063	0.108
X^2_{GHL}	0.062	0.108
X^2_{SW}	0.054	0.130
X^2_{SZ}	0.057	0.136

Using our notation, the Su-Wei (SW) test statistic is defined as

$$X^2_{\text{SW}} = \sup_{v \in \mathbb{R}^d} |\widetilde{R_n}(v)|,$$

where $v \in \mathbb{R}^d$,

$$\widetilde{R_n}(v) = \frac{1}{\sqrt{n}} \sum_{i=1}^{n} \mathbb{1}(X_i \leq v)[Y_i - m(\beta_n^\top X_i)],$$

and $\mathbb{1}(x \leq v)$ is an indicator for the event that each component of x is less than or equal to each respective component of v. With continuous covariates, finding the supremum can require $\widetilde{R_n}(v)$ to be evaluated at approximately n^{d-1} values of v. Adding to the complexity, obtaining p-values relies on a simulation procedure, described in more detail in Su and Wei (1991). Even for a relatively small sample size, such as $n = 100$, computing the SW test statistic and p-value can be computationally intensive with several predictors.

Stute and Zhu (2002) present a test statistic based on the Cramér-von Mises statistic applied to a transformed version of the R_n^1 process, $T_n R_n^1$. Setting x_0 to be, say, the 99th percentile of the observed linear predictors, $\beta_n^\top x_i$, $i = 1, \ldots, n$, the Stute-Zhu (SZ) test statistic can be defined as

$$X_{\mathrm{SZ}}^2 = \frac{1}{n \cdot \psi_n^2(x_0)} \sum_{i=1}^{n} \mathbb{1}(\beta_n^\top x_i \leq x_0)[T_n R_n^1(\beta_n^\top x_i)]^2 \cdot \sigma_n^2(\beta_n^\top x_i),$$

where

$$\psi_n(x_0) = \frac{1}{n} \sum_{i=1}^{n} \mathbb{1}(\beta_n^\top x_i \leq x_0)(Y_i - m(\beta_n^\top x_i))^2,$$

and $\sigma_n^2(u)$ is a consistent estimator of $\mathrm{Var}(Y \mid \beta_0^\top x = u)$, satisfying properties mentioned in their paper. The limiting sampling distribution of the test statistic is described in Stute and Zhu (2002).

Although they pose the null hypothesis in terms of the mean only and do not require a distributional assumption in the null hypothesis, we generally add such an assumption. A distributional assumption places restrictions on the forms of nuisance parameters in the SZ test statistic. For example, in the case of Poisson regression, $\sigma_n^2(u) = e^u$ for all n. We also modify the SZ test statistic in the Poisson model by having

$$\psi_n(x_0) = \frac{1}{n} \sum_{i=1}^{n} \mathbb{1}(\beta_n^\top x_i \leq x_0) m(\beta_n^\top x_i).$$

This modification allows the SZ test to detect violations of our narrower null hypothesis, such as overdispersion, that its original formulation would not permit, although even with this modification the ability of the SZ test to detect overdispersion is somewhat limited. For the selection of a kernel bandwidth in $T_n R_n^1$, we use a bandwidth of $0.5/\sqrt{n}$, which is used as part of a test in Stute et al. (1998).

We denote the percentile of the linear predictors used to define x_0 by p_{x_0}. We find that calculating $T_n R_n^1$ involves inverting matrices that might not be invertible even when p_{x_0} is much lower than 0.99, particularly when there are binary covariates or many variables present. We therefore try values of p_{x_0} in $\{0.99, 0.98, 0.95, 0.9, 0.85, 0.8, 0.75, 0.7\}$ in a decreasing order, and use the same value of p_{x_0} across all realizations in a given simulation setting. It is our opinion that having $p_{x_0} < 0.7$ does not include enough of the data for the test statistic to be truly meaningful, and in such cases we omit the calculation of the statistic unless otherwise stated.

With regards to the previous comment, the SZ test is not omitted in null setting 3, even though we were unable to perform some of the simulations even with $p_{x_0} = 0.7$. This can sometimes occur when binary covariates are present in the model because some matrices that should be inverted in the calculation of the test statistic become singular. Instead, data that resulted in an inability to compute the SZ test statistic was omitted, resulting in fewer than 2500 simulation realizations in each sub-setting. For the setting with larger models, the SZ test is not included because we observe that a large proportion of the data needs to be omitted when d is large and $n = 100$ for the test statistic to be computed. This is because at least $d - 1$ of the matrices that need to be inverted in the calculation of the SZ test statistic have less than full rank.

S.1.5 Application

We study the alcohol consumption dataset used in the study of DeHart et al. (2008), as described in Bilder and Loughin (2014). The goal of the study was to assess how the number of alcoholic drinks consumed is associated with factors such as self-esteem and negative romantic-relationship events. Bilder and Loughin (2014) performed a Poisson regression analysis on a subset of the data. The variable NUMALL, representing the number of alcoholic drinks consumed by a subject on their first Saturday, was regressed against several variables. After a fairly extensive analysis of the data, the authors arrived at a final model containing all of the main effects of the variables in Table S.6, including the following interactions: ROSN $\times$ PREL, AGE $\times$ ROSN, DESIRED $\times$ GENDER, DESIRED $\times$ AGE, and STATE $\times$ NEGEVENT.

Supplementary Table S.6: Description of variables used in the alcohol consumption study. Based on the subset of the data used in the Poisson regression analysis of Bilder and Loughin Bilder and Loughin (2014), each variable is derived from measurements for the first Saturday of each subject in the study.

Variable Name	Description
NUMALL (response)	Number of alcoholic drinks
NEGEVENT	Index for negative events
PREL	Index for positive romantic-relationship events
AGE	Age of the subject
ROSN	Long-term (trait) self-esteem level
STATE	Short-term (state) self-esteem level
GENDER	Gender of the subject
DESIRED	Desire of the subject to drink

Understanding the questionable validity of performing GOF tests following model selection on the same data, we test the fit of three different models using the GOF tests discussed in this paper. We have $n = 89$, and we use $G = 10$ and $G = 18$ groups. The larger number of groups is included to ensure that $G > d$, which is required for the naive HL test as was mentioned in Section 3, while still maintaining an average of about 5 observations per group. Because of the relatively high dimension of the data, the SW test is excluded in the evaluations of all three models.

We first examine the overall fit of the model mentioned at the beginning of this subsection (case 1). As seen in Table S.7, none of the tests considered reject the null hypothesis at the $\alpha = 0.05$ significance level. The naive HL gives somewhat larger p-values than the generalized version, matching expectations due to the moderately large number of variables relative to the sample size. For case 2 we fit a square root link instead of the original log link, retaining all of the variables from the model in case 1. The GHL results with both values of G and the naive HL with $G = 18$ suggest that the square root link may provide a poor fit. However, the naive HL with $G = 10$ and the SZ test do not reject the null hypothesis. Finally, in case 3, we omit all interactions from the the model in case 1. Here, we expect that the tests should detect a poor fit due to missing variables. In this case we see that the GHL test with 18 groups, the naive generalized HL test with 10 and 18 groups, and the SZ test reject the null hypothesis. While the GHL test with 10 groups fails to reject the null hypothesis that the model is correct, the p-value is still very close to 0.05. In general, tests based on grouped residuals, including our new proposed GHL test, seem to be quite sensitive to the number of groups used.

Supplementary Table S.7: GOF test results for various alcohol consumption models. Table C.6 from Klein and Moeschberger (1997) is used to approximate p-values for the SZ test.

Statistic	G	Case 1	Case 2	Case 3
X^2_{GHL}	10	0.433	*0.025*	0.050
$\widehat{C}^*_G$	10	0.675	0.275	*0.048*
X^2_{GHL}	18	0.065	*0.004*	*0.031*
$\widehat{C}^*_G$	18	0.118	*0.035*	*0.029*
X^2_{SZ}	—	0.101	0.988	*0.002*

S.2 Supplementary Material Appendix B: Discussion, Extensions, and Proofs

In this supplementary material we begin in Section S.2.1 by restating and proving Theorem 1 from the main paper. The restatement breaks the theorem into individual statements which are then proved in separate subsections. In Section S.2.2 we give conditions on an exponential family model which would imply conditions (A), (B), and (C). Section S.2.3 discusses how to check condition (D). In particular we prove that condition (D2) holds with $r \in \{G-1, G\}$ under reasonable conditions and show when the two possibilities arise. Section S.2.4 gives the precise conditions on the alternative distribution under which we have established the consistency of our test. In that section we state and prove a precise version of the result. Finally, because the proofs rely on some empirical process results, in Section S.2.5 we state and prove the precise versions we need, drawing on Kosorok (2007).

S.2.1 Proof of Theorem 1

Theorem 1 is phrased in terms of assumptions based on the work of Stute (1997). That work describes the behaviour of the process R_n^1 defined in (6) using assumptions weaker than requiring the conditional density of Y given X to come from the exponential family density (2) or the exponential dispersion density (3). The Stute (1997) conditions are enough to deduce asymptotic normality of the vector S_n^1 defined in (7), which is the first conclusion of Theorem 1. The result on the limiting χ^2 distribution of our statistic requires extra moment conditions beyond those of Stute (1997); these extra conditions account for the length of condition (B). Finally, Theorem 1 asserts that the extra assumption that the conditional density of Y given X does come from the exponential family density (2) or the exponential dispersion density (3) implies some of the conditions.

S.2.1.1 Restatement of Theorem 1

We now restate Theorem 1 to facilitate the proof and to make it easier for readers to see which assumptions are important for which precise conclusions. Suppose $(X_1, Y_1), (X_2, Y_2), \cdots$ are independent and identically distributed random variables each with the same distribution as (X, Y) where X takes values in $\mathbb{R}^d$ and Y is real valued. Assume

$$\mathrm{E}(Y^2) < \infty.$$

Let m and v be given functions. Let $\mathbf{B}_0 = \{\beta : P(m(\beta^\top X) \in b'(\Theta)) = 1\}$. Assume $\mathbf{B}_0$ has a non-empty interior, $\mathrm{int}(\mathbf{B}_0)$. Assume that there is a $\beta_0 \in \mathrm{int}(\mathbf{B}_0)$ and a $\phi_0 > 0$ such that

$$\mathrm{E}(Y|X) = m(\beta_0^\top X) \tag{S.1}$$

almost surely and

$$\mathrm{Var}(Y|X) = \phi_0 \cdot v(m(\beta_0^\top X)) \tag{S.2}$$

almost surely.

The parameter β is estimated by solving the likelihood equations corresponding to (2). The score function for such a model is

$$U(\beta) = \sum_{i=1}^{n} U_i(\beta) = \sum_{i=1}^{n} \frac{Y_i - m(\beta^\top X_i)}{v(m(\beta^\top X_i))} m'(\beta^\top X_i) X_i. \tag{S.3}$$

The β component of the score function for the exponential dispersion model (3) is

$$U(\beta)/\phi.$$

These forms are a consequence of the fact that in the exponential density (2) we have

$$\mathrm{E}_\theta(Y) = b'(\theta)$$

and

$$\mathrm{Var}(Y) = b''(\theta).$$

Thus, under the exponential family model for the conditional mean of Y given X we have

$$\theta = (b')^{-1}(m(\beta^\top X))$$

and

$$\mathrm{Var}(Y|X) = b'' \left\{ (b')^{-1}(m(\beta^\top X)) \right\}.$$

Therefore, under the exponential family model (2) the function v is simply

$$v(m) = b'' \left\{ (b')^{-1}(m) \right\}.$$

For the exponential dispersion form we have

$$\mathrm{Var}(Y|X) = \phi_0 \cdot v(m(\beta_0^\top X)).$$

The likelihood equations for β are usually solved by Iteratively Reweighted Least Squares; these equations form a set of unbiased estimating equations under the assumptions above.

Our first set of conditions, adapted from Stute (1997), are:

Condition (A)

(i) The matrix

$$I_1(\beta_0) \equiv \mathrm{E}\left[X X^\top \frac{\{m'(\beta_0^\top X)\}^2}{v\left(m(\beta_0^\top X)\right)} \right]$$

exists and is positive definite.

(ii) Let $\ell(X_i, Y_i, \beta_0) = [I_1(\beta_0)]^{-1} U_i(\beta_0)$. Under the null hypothesis, we have

$$n^{1/2}\{\beta_n - \beta_0\} = n^{-1/2} \sum_{i=1}^{n} \ell(X_i, Y_i, \beta_0) + o_P(1).$$

Condition (B1): The function m is twice continuously differentiable and the function v is continuously differentiable. For some $\delta > 0$ we have

$$\mathrm{E}\left[\sup_{\{\beta : \|\beta - \beta_0\| \le \delta\}} \{\max_j |X_j \ m'(\beta^\top X)|\} \right] < \infty. \tag{S.4}$$

Condition (C): Define, for $u \in \mathbb{R}$ $\tilde{H}(u, \beta) = \mathrm{E}\left\{\mathrm{Var}(Y|X)\mathbb{1}(\beta^\top X \le u)\right\}$. Then $\tilde{H}$ is uniformly continuous in u at β_0.

We require stronger conditions to deduce the chi-square limiting distribution of our statistic. We need, from the main text, condition (D) and the extra moment conditions given in condition (B).

Here is our restatement of Theorem 1 from the main paper.

Theorem S.1. *Suppose that* $\mathrm{E}(Y^2) < \infty$*. Assume the cell boundaries* $k_{n,g}$ *satisfy* $k_{n,g} \xrightarrow{p} k_g$ *for* $g = 0, \ldots, G$ *and that the* k_g *are distinct. Assume that* β_0 *belongs to the interior of* B_0*. Then:*

1. *Under conditions (A), (B1), and (C) we have*

$$S_n^1 \xrightarrow{d} MVN_G(0, \phi_0 \Sigma) \equiv S_\infty^1,$$

where S_n^1 *is as defined in (7), and* Σ *is given by (13).*

2. *Assume conditions (A), (B), and (C) hold. For any sequence of matrices* Σ_n *satisfying condition (D), and any sequence of estimates* ϕ_n *converging to* ϕ_0 *then, putting* $r = \mathrm{rank}(\Sigma)$,

$$S_n^{1\top} \Sigma_n^{+} S_n^1 / \phi_n \xrightarrow{d} \chi_r^2.$$

3. *Under conditions (A), (B), and (C), the matrix* Σ_n *in (10) converges almost surely to* Σ*; in particular, condition (D1) holds.*

4. *If conditions (i) and (ii) of Section 3.4 are satisfied, then conditions (B1) and (C) hold.*

S.2.1.2 Proof of Statement 1

We borrow and modify some of the notation used in Stute and Zhu (2002). Under our conditions they show that the sequence of processes R_n^1, where, for $u \in \mathbb{R}$,

$$R_n^1(u) = \frac{1}{\sqrt{n}} \sum_{i=1}^{n} \mathbb{1}(\beta_n^\top X_i \le u)[Y_i - m(\beta_n^\top X_i)], \tag{S.5}$$

converges weakly to a centered Gaussian process R^1_∞ with continuous sample paths. This process has the structure

$$R^1_\infty(u) = R_\infty(u) - Q^\top(u)\Gamma,$$

whose terms we now describe. The process R_∞ is a centered Gaussian process with continuous sample paths and covariance kernel $K(s,t) = \psi(\min\{s,t\})$, where, for $w \in \mathbb{R}$

$$\psi(w) = \mathrm{E}\left\{\mathrm{Var}(Y|\beta_0^\top X)\mathbb{1}(\beta_0^\top X \le w)\right\} = \int_{-\infty}^{w} \mathrm{Var}(Y|\beta_0^\top X = u)F_{\beta_0}(du).$$

Here F_{β_0} denotes the distribution of $\beta_0^\top X$.

Define the column vector

$$q(x,\beta) = \frac{\partial m(\beta^\top x)}{\partial \beta} = (q_1(x,\beta),\ldots,q_d(x,\beta))^\top = m'(\beta^\top x)x.$$

Stute and Zhu (2002) define, for $u \in \mathbb{R}$, the vector-valued function $Q(u)$ with components $Q_i(u), i = 1\ldots,d$ by

$$Q(u) \equiv Q(u,\beta_0) = \mathrm{E}(q(X,\beta_0)\mathbb{1}(\beta_0^\top X \le u));$$

they show that each Q_i is continuous in u at β_0. Finally, Γ is a d-dimensional multivariate normal vector with zero means and covariance matrix $[I_1(\beta_0)]^{-1}$, the inverse of the Fisher information matrix for a single observation. From Stute and Zhu (2002),

$$\mathrm{Cov}(R_\infty(u), Q^\top(s,\beta_0)\Gamma) = Q^\top(s,\beta_0)\,\mathrm{E}\left[\mathbb{1}(\beta_0^\top X \le u)\left\{Y - m(\beta_0^\top X)\right\}\ell(X,Y,\beta_0))\right].$$

Because the process R^1_∞ has continuous sample paths, for interval endpoints $k_{n,g}$ converging to the non-random interval endpoints, k_g, we have the conclusion

$$S^1_n \xrightarrow{d} \left\{(R_\infty(k_g) - Q^\top(k_g)\Gamma) - (R_\infty(k_{g-1}) - Q^\top(k_{g-1})\Gamma)\right\}_{g=1}^{G} \equiv S^1_\infty,$$

where S^1_∞ is multivariate normal, $\mathrm{MVN}_G(0,\Sigma)$ and Σ is as defined in (13) from the main text.

This completes the proof of Statement 1 of Theorem 1.

S.2.1.3 Proof of Statement 2

Statement 2 follows from Theorem 1 of Andrews (1987) which we now put in our notation. They study quadratic forms $U_n^\top A_n^+ U_n$. Andrews' first condition is that

$$U_n \xrightarrow{d} \mathrm{MVN}_G(0, A)$$

for some symmetric, non-negative matrix A, whose rank we denote by r. We established this hypothesis above for our quadratic form with $A = \Sigma$. Andrews' second condition is that

$$A_n \xrightarrow{p} A.$$

This is condition (D1) which we are assuming for the matrix Σ_n in our second statement. Conclusion (a) of Andrews' Theorem 1 is that under these two conditions we have

$$U_n^\top A_n^+ U_n \xrightarrow{d} \chi_r^2,$$

if a quantity that Andrews denotes by Q_n converges to 0 in probability. However, Andrews observes (on page 352 in the second sentence of the bottom paragraph) that if $\text{rank}(A_n) \leq \text{rank}(A)$ then $Q = 0$. In a comment immediately below Theorem 1, Andrews observes that if his first two conditions given above hold and $\text{rank}(A_n) \xrightarrow{p} \text{rank}(A)$ then the chi-squared limit above also holds. Because we have assumed Σ_n satisfies condition (D2), this proves Statement 2.

S.2.1.4 Proof of Statement 3

We are to prove, under conditions (A), (B) (from the main text) and (C) that $\Sigma_n \xrightarrow{p} \Sigma$. This will be a corollary to Theorem S.2, which we prove below. Theorem S.2 is a somewhat more general result that will be useful in our study of consistency properties of our test and our results on the rank of Σ. We will see below that the estimate Σ_n of the matrix Σ depends on the distribution of X and on the modeled conditional mean and variance of Y given X (that is, on the functions m and v) as well as the estimates β_n and ϕ_n. It does not depend directly on Y—only indirectly through the estimates and perhaps the cell boundaries.

Consider a pair β, ϕ of parameter values, a distribution of the covariates X, and a set of non-random cell boundaries $k_0 = -\infty < k_1 < k_2 < \cdots k_{G-1} < k_g = \infty$, where $\phi > 0$ and β is such that

$$\mathrm{E}\left[|m'(\beta^\top X)| \|X\|)\right] < \infty. \tag{S.6}$$

$$\mathrm{E}\left[\|X\|^2 \frac{\left\{m'(\beta^\top X)\right\}^2}{v(m(\beta^\top X))}\right] < \infty, \text{ and} \tag{S.7}$$

$$\mathrm{E}\left[v(m(\beta^\top X))\right] < \infty. \tag{S.8}$$

We may then define the following quantities. Define the $G \times d$ matrix Δ with g, jth entry ($j \in \{1, \cdots, d\}$)

$$\Delta_{gj}(\beta, k) = \mathrm{E}\left[m'(\beta^\top X) X_j \mathbb{1}(k_{g-1} < \beta^\top X \leq k_g)\right].$$

Define

$$I^*(\beta) = \mathrm{E}\left[XX^\top \frac{\left\{m'(\beta^\top X)\right\}^2}{v(m(\beta^\top X))}\right].$$

Let $\Sigma^{(1)}(\beta)$ be the $G \times G$ diagonal matrix whose gth diagonal entry is

$$\mathrm{E}\left[v(m(\beta^\top X))\mathbb{1}(k_{g-1} < \beta^\top X \leq k_g)\right]$$

Let $\Sigma^{(2)}(\beta)$ be the $G \times G$ matrix

$$\Delta I^*(\beta)^{-1} \Delta^\top.$$

Finally, define

$$\Sigma(\beta, \phi) = \phi \left\{ \Sigma^{(1)}(\beta) - \Sigma^{(2)}(\beta) \right\} \equiv \phi \Sigma(\beta).$$

It may easily be checked that this definition matches the formula for Σ given in the main text when $\beta = \beta_0$ and condition (B1) of Theorem S.1 holds. Condition A and the remaining parts of condition B imply the moment conditions (S.6), (S.7), and (S.8) hold for $\beta = \beta_0$.

We now show that for any sequence of random coefficient vectors, say $\tilde{\beta}_n$, converging to some β^* we have

$$\Sigma_n(\tilde{\beta}_n) \xrightarrow{p} \Sigma(\beta^*),$$

under some moment conditions which are slightly stronger than those used previously.

To state the conditions, we define

$$\begin{aligned}
w_A(u) &= \frac{d}{du} v(m(u)) = m'(u) v'(m(u)), \\
w_B(u) &= m''(u), \text{ and} \\
w_C(u) &= \frac{d}{du} \frac{(m'(u))^2}{v(m(u))}.
\end{aligned}$$

Then, for $\delta > 0$, define

$$\begin{aligned}
M_{A,\beta^*,\delta}(x) &= \|x\| \sup\{|w_A(\beta^\top x)|, \|\beta - \beta^*\| \leq \delta\}, \\
M_{B,\beta^*,\delta}(x) &= \|x\|^2 \sup\{|w_B(\beta^\top x)|, \|\beta - \beta^*\| \leq \delta\}, \text{ and} \\
M_{C,\beta^*,\delta}(x) &= \|x\|^3 \sup\left\{|w_C(\beta^\top x)|, \|\beta - \beta^*\| \leq \delta\right\}.
\end{aligned}$$

We now introduce our stronger moment conditions. Condition (E), which follows, extends condition (B).

Condition (E(β^*))

There is a $\delta > 0$ such that

$$\mathrm{E}\left\{M^2_{A,\beta^*,\delta}(X)\right\} < \infty,$$

$$\mathrm{E}\left\{M^2_{B,\beta^*,\delta}(X)\right\} < \infty,$$

and

$$\mathrm{E}\left\{M_{C,\beta^*,\delta}(X)\right\} < \infty.$$

Moreover:

$$\mathrm{E}\left[v^2(m(\beta^{*\top} X))\right] < \infty,$$

$$\mathrm{E}\left[\left\{m'(\beta^{*\top} X)\right\}^2 \|X\|^2\right] < \infty,$$

and

$$\mathrm{E}\left[\frac{\left\{m'(\beta^{*\top}X)\right\}^2}{v(m(\beta^{*\top}X))}\|X\|^2\right] < \infty.$$

Below we write condition (E) for condition (E(β_0)), the crucial special case where the null hypothesis holds and β^* is the true parameter vector.

In order to state the following theorem carefully we will emphasize the dependence of the covariance matrix Σ on both β and a limiting set of cell boundaries $k = (k_0, \ldots, k_g)$. Correspondingly we will indicate the dependence of the estimate Σ_n on a parameter vector β (which will not necessarily be the MLE β_n) and cell boundaries $k_n = (k_{n,0}, \ldots, k_{n,G})$. That is, we write $\Sigma(\beta, k)$ and $\Sigma_n(\beta, k_n)$. Let $\mathcal{K}$ be the set of all $k = (k_0, \ldots, k_G)$ with $k_0 < k_1 \cdots < k_G$.

Theorem S.2. *Assume condition (A). Assume that the distribution of X satisfies condition (C). Let $\tilde{\beta}_n$ be a sequence of random vectors converging in probability to some β^*. Let k_n denote a possibly random sequence of cell boundaries converging in probability to $k \in \mathcal{K}$. Assume that condition (E(β^*)) holds. With $\delta > 0$ as given by condition (E(β^*)) define, for any $0 < \kappa < \delta$ the ball $N_\kappa = \{\beta : \|\beta - \beta^*\| \leq \kappa < \delta\}$. Then,*

1. *Almost surely, for every $\kappa < \delta$,*

$$\Sigma_n(\beta, k_n) \to \Sigma(\beta, k),$$

   uniformly for $\beta \in N_\kappa$ and $k \in \mathcal{K}$.

2. *The matrix $\Sigma(\beta, k)$ depends continuously on β, k on the set $N_r \times \mathcal{K}$.*

3. *The sequence of matrices $\Sigma_n(\tilde{\beta}_n, k_n)$ converges in probability to $\Sigma(\beta^*, k)$.*

Corollary 1. *Under the conditions of Theorem S.2, if $\tilde{\phi}_n$ is any sequence of random positive scalars converging to some ϕ^* in probability, then*

$$\Sigma_n(\tilde{\beta}_n, k_n, \tilde{\phi}_n) = \tilde{\phi}_n \Sigma_n(\tilde{\beta}_n, k_n) \to \phi^* \Sigma(\beta^*, k)$$

in probability.

The most important case of this theorem arises when the null hypothesis holds, $\tilde{\beta}_n$ is the MLE, β_n, and β^* is the true parameter value β_0. In that case Theorem S.2 becomes:

Corollary 2. *Under conditions (A), (B), and (C), our estimator Σ_n, given by (10), is consistent for Σ under the null hypothesis. That is, condition (D1) is satisfied.*

Proof of Theorem S.2 Define the $G \times n$ matrix-valued function, $G_n(\beta)$, by

$$(G_n(\beta, k_n))_{gi} = \mathbb{1}(k_{n,g-1} < \beta^\top X_i \leq k_{n,g}),$$

for $i = 1, \ldots, n$, and $g = 1, \ldots, G$. Similarly, define the $n \times n$ matrices

$$V_n^{1/2}(\beta) = \text{diag}\left(\left[v(m(\beta^\top X_i))\right]^{1/2}\right)$$
$$W_n^{1/2}(\beta) = \text{diag}\left(\frac{m'(\beta^\top x_i)}{v(m(\beta^\top x_i))^{1/2}}\right).$$

The estimator $\Sigma_n(\tilde{\beta}_n, k_n)$ can be written in the form

$$A_n(\tilde{\beta}_n, k_n) - B_n(\tilde{\beta}_n, k_n)C_n^{-1}(\tilde{\beta}_n)B_n^\top(\tilde{\beta}_n, k_n),$$

where $A_n(\tilde{\beta}, k_n)$ is a $G \times G$ matrix, $B_n(\tilde{\beta}, k_n)$ is a $G \times d$ matrix, and $C_n(\tilde{\beta})$ is $d \times d$. These matrices are given by

$$A_n(\tilde{\beta}, k_n) = \frac{1}{n} G_n(\tilde{\beta}, k_n) V_n(\tilde{\beta}) G_n(\tilde{\beta}, k_n)^\top,$$
$$B_n(\tilde{\beta}, k_n) = \frac{1}{n} G_n(\tilde{\beta}, k_n) V_n^{1/2}(\tilde{\beta}) W_n^{1/2}(\tilde{\beta}) X^*, \text{ and}$$
$$C_n(\tilde{\beta}) = \frac{1}{n} X^{*\top} W_n(\tilde{\beta}) X^*.$$

The matrices $G_n(\tilde{\beta}, k_n)$, $V_n(\tilde{\beta})$, and $W_n(\tilde{\beta})$ are the same as G_n, V, and W defined in the main text, except that $\tilde{\beta}_n$ replaces both β_0 and β_n in the definitions. Here we have emphasized the dependence of each entry on $\tilde{\beta}$; we will show that under our conditions each of A_n, B_n, and C_n converges to its expected value uniformly in $(\beta, k) \in \overline{\mathcal{N}} \times \mathcal{K}$ where $\overline{\mathcal{N}} = \{\beta : \|\beta - \beta_0\| \leq \delta\}$ with δ from condition (E). We will also show those limits are continuous functions of β. This will finish our proof of consistency.

Each of these three matrices can be written in the form

$$\frac{1}{n} \sum_{i=1}^{n} H(X_i, \beta).$$

Under our assumptions, the matrix valued functions H involved have finite expectations for all $\beta \in \overline{\mathcal{N}}$. Our proof then uses Glivenko-Cantelli theorems, that is, uniform laws of large numbers; see Lemmas 1 and 2 below.

Consistency of A_n

For the matrix A_n the g, g' entry in $H(x, \beta)$ is

$$\mathbb{1}(k_{g-1} < \beta^\top x \leq k_g) v(m(\beta^\top x)) \mathbb{1}(k_{g'-1} < \beta^\top x \leq k_{g'}),$$

which vanishes unless $g = g'$, in which case it is simply

$$\mathbb{1}(k_{g-1} < \beta^\top x \leq k_g) v(m(\beta^\top x)).$$

We apply Lemma 2. For $u \in \mathbb{R}$ and $\beta \in \mathbb{R}^d$ define the function $f_{\beta,u}$ by

$$f_{\beta,u}(x) = v(m(\beta^\top x))\mathbb{1}(\beta^\top x \le u),$$

and the class of functions $\mathcal{F}_A$ by

$$\mathcal{F}_A = \{f_{\beta,u} : \|\beta - \beta_0\| \le \delta, u \in \mathbb{R}\}.$$

We apply Lemma 2 with $\beta^* = \beta_0$, $h(\beta, x) = v(m(\beta^\top x))$, and $M = M_{A,\beta^*,\delta}$.

With these choices, conditions i-iii of the Lemma come immediately from condition (E). We conclude that $\mathcal{F}_A$ is P-Glivenko-Cantelli. That is,

$$\sup\left\{\left|\frac{1}{n}\sum_{i=1}^n f_{\beta,u}(X_i) - \mathrm{E}(v(m(\beta^\top X))\mathbb{1}(\beta^\top X \le u))\right| ; \beta \in \overline{\mathcal{N}}, u \in \mathbb{R}\right\} \to 0$$

almost surely. Let

$$J(\beta, u) = \mathrm{E}\left\{v(m(\beta^\top X))\mathbb{1}(\beta^\top X \le u)\right\},$$

and

$$J_n(\beta, u) = \frac{1}{n}\sum_{i=1}^n v(m(\beta^\top X_i))\mathbb{1}(\beta^\top X_i \le u).$$

The g, g entry in A_n is

$$J_n(\beta_n, k_{n,g}) - J_n(\beta_n, k_{n,g-1}).$$

We have shown that, uniformly over $(\beta, k) \in \overline{\mathcal{N}} \times \mathcal{K}$ we have

$$\{J_n(\beta, k_g) - J_n(\beta, k_{g-1})\} - \{J(\beta, k_g) - J(\beta, k_{g-1})\} \to 0$$

almost surely. Consistency of A_n then follows from continuity in β, k of $J(\beta, k)$ which we now establish. The dominated convergence theorem shows that for any deterministic sequence β_n converging to β_0 we have

$$\lim_{n\to\infty} \mathrm{E}\left\{h(\beta_n, X)\mathbb{1}(\beta_n^\top X \le k_g)\right\} = \mathrm{E}\left\{h(\beta_0, X)\mathbb{1}(\beta_0^\top X \le k_g)\right\},$$

provided $P(\beta_0^\top X = k_g) = 0$. This last follows from condition (C).

Consistency of B_n

For the matrix B_n, the g, j entry in $H(x_i, \beta)$ is

$$\mathbb{1}(k_{g-1} < \beta^\top x_i \le k_g)m'(\beta^\top x_i)x_{ij}.$$

We define, for $u \in \mathbb{R}$, $\beta \in \mathbb{R}^d$, and $j \in \{1, \ldots, d\}$, the function $f_{\beta,u,j}$ to be the jth component of

$$f_{\beta,u}(x) = m'(\beta^\top x)\mathbb{1}(\beta^\top x \le u)x.$$

The argument for A_n together with the assumption on $M_{B,\beta^*,\delta}$ may be followed to prove that $B_n(\beta_n, k_n)$ converges almost surely to $\Delta(\beta, k_\infty)$.

Consistency of C_n

Finally, we consider the matrix C_n and show that $C_n(\beta_n)$ is a consistent estimator of the Fisher information matrix for a single observation. This matrix has the form

$$C_n(\beta) = \frac{1}{n}\sum_{i=1}^{n} X_i X_i^\top \frac{(m'(\beta^\top X_i))^2}{v(m(\beta^\top X_i))}.$$

Define

$$J_C(\beta) = \mathrm{E}\left(XX^\top \frac{(m'(\beta^\top X))^2}{v(m(\beta^\top X))}\right),$$

and observe that $J_C(\beta_0) = I_1(\beta_0)$, the Fisher Information matrix. (It is a consequence of this observation and condition (E) that $J_C(\beta)$ is finite for each $\beta \in \overline{\mathcal{N}}$.) We now apply Lemma 1; the Lemma is applied to the j, j' component of C_n but we can use the same bounding function for all components. In the Lemma we take $h(\beta, x)$ to be the j, j' entry in

$$xx^\top \frac{(m'(\beta^\top x))^2}{v(m(\beta^\top x))},$$

and $M(x) = M_{C,\beta^*\delta}(x)$.

S.2.1.5 Proof of Statement 4

Assumption (ii) of Section 3.4 includes the statement that the support of X is compact which makes all the moment conditions easy; this establishes condition (B). Assumption (ii) also includes the assertion that the law of $\beta^\top X$ is absolutely continuous with a bounded Lebesgue density for all β in a neighbourhood of β_0; this clearly implies condition (C).

S.2.2 Verifying Conditions (A), (B), and (C) for Various GLMs

In this section we discuss how to verify the conditions (A), (B), and (C) when we assume the response variables follow the model (2). For specific GLM families and a variety of common link functions we translate our conditions into simpler terms. We consider the following choices of pairs m, v: the normal family with identity link and $v \equiv 1$; the Poisson model with log or square root link and $v(m) = m$; the Bernoulli model with m being a cumulative distribution and $v(m) = m(1-m)$; the Gamma model with log link and $v(m) = m^2$; the inverse Gaussian with log link and $v(m) = m^3$, and the Negative Binomial with $v(m) = m + m^2/\lambda$ – we assume λ is known.

S.2.2.1 Condition (A)

Conditions for asymptotic normality and weak consistency of the MLE β_n for β_0 in GLMs can be found in Fahrmeir and Kaufmann (1985). They show that in a GLM *without* a dispersion parameter and with the canonical link function there is, under very mild conditions, a root β_n of the likelihood equations that is consistent for β_0 in the case of iid covariates.

We first consider canonical (natural) link functions, since such links make the log-likelihood convex. If X has compact support and $\mathrm{E}(XX^\top)$ is positive definite (i.e., the covariance matrix of X is positive definite), then β_n asymptotically exists and is strongly consistent, by Corollary 3 of Fahrmeir and Kaufmann (1985). We now consider the case where the support of X is not compact and introduce condition (R_s) of Fahrmeir and Kaufmann (1985):

$(R_s)(i) : I_1(\beta_0)$ exists and is positive definite, and

$(R_s)(ii) : \mathrm{E}\left[\max_{\beta \in N} XX^\top v\left\{m\left(\beta^\top X\right)\right\}\right]$ exists for some compact neighbourhood, N, of β_0.

If condition (R_s) holds, then β_n asymptotically exists and is strongly consistent, also by their Corollary 3. The expansion in condition (A) follows.

For link functions other than the canonical link, it can be the case that the score function has multiple roots. In this case, results from Fahrmeir and Kaufmann (1985, 1986) assert that there is a consistent root and that this root satisfies the expansion in condition (A). Some additional conditions might be required to establish weak consistency of β_n with noncanonical links.

S.2.2.2 Condition (B)

The inverse link functions considered are mentioned in Section 3.4 of the paper. All of these are continuously differentiable on the whole real line. Let Θ be the set of θ for which $\int \exp(y\theta)\nu(dy) < \infty$. Then Θ is an interval in the real line. On the interior of that interval the function b has infinitely many derivatives and is strictly convex so in particular b, b' and b'' are all continuous on the interior of Θ. The function b' is strictly monotone and has an infinitely differentiable inverse. Continuity of the map $u \mapsto v(m(u))$ thus holds, since

$$v(m(u)) = b''(\theta) = b''(b'^{-1}(m(u)))).$$

Thus the first part of condition (B) is met.

The second part of condition (B) imposes 6 moment conditions on the covariates. We consider the 6 conditions in turn. Since conditions (B1), (B4), and (B5) depend only on the choice of inverse link function m we do them first. The other 3 conditions depend on the combination of v and m.

To verify condition (B1) it is enough to prove that there is a function $M(x)$ such that

$$\mathrm{E}(M(X)) < \infty.$$

and

$$\sup_{\{\beta:\|\beta-\beta_0\|\le\delta\}} \{\max_j |X_j\ m'(\beta^\top X)| \le M(X)$$

almost surely. Consider first the log link, for which $m(\mu) = e^\mu$. If there is a neighbourhood of β_0 in which X has a moment generating function then condition (B1) holds. For the logit, probit, cauchit, cloglog, and identity links it is enough that X have a finite mean; that is, for each $1 \le j \le d$ we have $\mathrm{E}(|X_j|) < \infty$. For the square root link we require finite variances: for each j we require $\mathrm{E}(X_j^2) < \infty$. All of these moment conditions are immediate consequences of an overall assumption that the covariates X lie in some bounded set with probability 1. Here are some details.

Consider the log link, i.e., $m(\beta^\top x) = \exp(\beta^\top x)$. Suppose that for some $\kappa > 0$ the random vector X has a finite moment generating function $\mathrm{E}(\exp(\beta^\top X)) < \infty$ for all β such that $\|\beta - \beta_0\| \le \kappa$. Then it can be shown using the convexity of $\beta \mapsto \exp(\beta^\top x)$ that for any $0 < \delta < \kappa$ and any $c > 0$ we have

$$\mathrm{E}\left[\sup_{\{\beta:\|\beta-\beta_0\|\le\delta\}} \|X\|^c \exp\{\beta^\top X\}\right] < \infty.$$

Our sufficient condition for (B1) given above then holds with

$$M(x) = \|x\| \exp\{(\|\beta_0\| + \delta)\|x\|\}.$$

Indeed, for $j = 1, \ldots, d$, we have, for all β with $\|\beta - \beta_0\| \le \delta$,

$$\begin{aligned} |q_j(x,\beta)| &= \left|m'\left(\beta^\top x\right) x_j\right| \\ &= \left|x_j \exp(\beta^\top x)\right| \\ &\le M(x). \end{aligned}$$

For the identity link we have $q_j(x,\beta) = x_j$. For the four bounded links the derivative m' is also bounded and we have

$$|q_j(x,\beta)| \le \|x\|\|m'\|_\infty.$$

For the square root link we see $m'(u) = 2u$ and

$$|q_j(x,\beta)| \le 2\|x\| \sup_{\{\beta:\|\beta-\beta_0\|\le\delta\}} |\beta^\top x| \le 2\|x\|^2 \sup_{\{\beta:\|\beta-\beta_0\|\le\delta\}} \|\beta\|^2.$$

Thus for this link, and any of our models we need finite second moments of the covariates to deduce condition (B1).

It remains to interpret condition (B) for each combination of inverse link m and each variance function v considered in this paper. We run through our models, which determine v, and then consider those m which might be used for that model. We first present a table of link and variance combinations, along with the functions appearing in our conditions.

Supplementary Table S.8: Several distribution and link function combinations.

Distribution	$m(u)$	$v(m)$	$m'(u)$	$m'(u)v'(m(u))$	$m''(u)$	$(m'(u))^2/v(m(u))$
Normal(μ, σ^2)	u	1	1	0	0	1
Poisson(λ)	e^u	m	e^u	e^u	e^u	e^u
Poisson(λ)	u^2	m	$2u$	$2u$	2	4
Bernoulli(π)	$F(u)$	$m(1-m)$	$f(u)$	$(1-2F(u))f(u)$	$f'(u)$	$f^2(u)/[F(u)(1-F(u))]$
Gamma(μ, k)	e^u	m^2	e^u	$2e^{2u}$	e^u	1
IG(μ, λ)	e^u	m^3	e^u	$3e^{3u}$	e^u	$1/e^u$
NB(μ, k)	e^u	$m + m^2/k$	e^u	$e^u + 2e^{2u}/k$	e^u	$e^u/(1+e^u/k)$

Normal Family: Here $v(m) \equiv 1$ and we may take $M_{A,\beta_0,\delta}(x) = M = 0$ for any link and any $\delta > 0$. The most common link for the normal family is the identity for which $m' \equiv 1$, $m'' \equiv 0$, and $M_{B,\beta_0,\delta} = 0$. In this case condition (B) reduces to assuming that the covariates have finite second moments. The conditions on $v(m(\beta_0^\top X))$ and on $m'(\beta_0^\top X)$ are both trivial.

Poisson Family: Here $v(m) = m$. Common links are the log link ($m(u) = e^u$) and the square root link ($m(u) = u^2$).

For the log link we find that $v(m(u)) = e^u$ so that

$$M_{A,\beta_0,\delta}(x) = \|x\| \sup\{e^{\beta^\top x} : \|\beta - \beta_0\| \leq \delta\},$$

which means our condition is that X has a finite moment generating function in a neighbourhood of $2\beta_0$. We also have

$$m''(u) = m'(u) = e^u,$$

and

$$M_{B,\beta_0,\delta}(x) = \|x\|^2 \sup\{e^{\beta^\top x} : \|\beta - \beta_0\| \leq \delta\}.$$

The condition is the same as the one associated with $M_{A,\beta_0,\delta}$. Finally,

$$\frac{d}{du} \frac{(m'(u))^2}{v(m(u))} = e^u,$$

so our condition is that X has a finite moment generating function in a neighbourhood of β_0. We also need $\mathrm{E}(e^{2\beta_0^\top X}) < \infty$ and $\mathrm{E}(e^{2\beta_0^\top X}\|X\|^2) < \infty$. These lead to the same condition as the one associated with both $M_{A,\beta_0,\delta}$ and $M_{B,\beta_0,\delta}$. That is: for the Poisson family with log link, condition (B) holds as long as X has a finite moment generating function in a neighbourhood of $2\beta_0$.

For the square root link we have $m(u) = v(m(u)) = u^2$. We find that

$$w_A(u) = 2u.$$

Thus,

$$M_{A,\beta_0,\delta}(x) = 2\|x\| \sup\{|\beta^\top x| : \|\beta - \beta_0\| \le \delta\}.$$

Writing β in the form $\beta_0 + ax + bz$ with z any vector perpendicular to x shows that we can regard $b = 0$, and then

$$M_{A,\beta_0,\delta}(x) = 2\|x\| \sup\{|\beta_0^\top x| + a\|x\|^2 : |a|\|x\| \le \delta\}.$$

The maximum of this piecewise linear function of a that occurs in the braces must occur on the boundary of the interval imposed on a, so it is easily checked that

$$M_{A,\beta_0,\delta}(x) = 2\|x\| \left(|\beta_0^\top x| + \delta\|x\|\right).$$

Thus, $M_{A,\beta_0,\delta}$ is square integrable if X has 4 finite moments. We see that

$$m''(u) = 2,$$

and thus

$$M_{B,\beta_0,\delta}(x) = 2\|x\|^2,$$

which requires 4 finite moments. Finally, it is easily checked that for this link

$$C_n(\beta) = \frac{4}{n}\sum_{i=1}^{n} X_i X_i^\top,$$

which does not depend on β. For the Poisson family with a square root link, we also require $\mathrm{E}((\beta_0^\top X)^4) < \infty$ and $\mathrm{E}(4(\beta_0^\top X)^2\|X\|^2) < \infty$, but these do not add any further moment conditions. That is: for the Poisson family with square root link, condition (E) holds as long as X has 4 finite moments.

Bernoulli Family: For the Bernoulli(θ) model we have $v(m) = m(1-m)$. The inverse links we consider all have the form

$$m(u) = F(u),$$

for a smooth cdf F with corresponding smooth density f. We therefore have

$$v(m(u)) = F(u)(1 - F(u)),$$

which is bounded by 1, and

$$m'(u) = f(u).$$

For all 4 links (logit, probit, cauchit, and cloglog) we find that there is a constant, C, such that, for all u,

$$f(u) \leq C.$$

For all 4 links there is also another constant, C_2, such that, for all u,

$$|f'(u)| \leq C_2.$$

Therefore,

$$|w_A(u)| = |f(u)(1 - 2F(u))| \leq C,$$

and so

$$M_{A,\beta_0,\delta}(x) \leq \|x\|C,$$

which amounts to 2 finite moments. Also,

$$M_{B,\beta_0,\delta}(x) \leq C_2\|x\|^2,$$

which amounts to 4 finite moments.

The quantity

$$|w_C(u)| = \left| \frac{d}{du} \frac{(m'(u))^2}{v(m(u))} \right| = \left| \frac{2f(u)f'(u)}{F(u)(1 - F(u))} - \frac{f^3(u)(1 - 2F(u))}{\left[F(u)\left\{1 - F(u)\right\}\right]^2} \right|$$

is bounded by some constant. Therefore, our condition on $M_{C,\beta_0,\delta}(x)$ amounts to requiring three finite moments for X.

Gamma Family: Here $v(m) = m^2$. We consider the log link, $m(u) = e^u$. The square root link, $m(u) = u^2$, is included to highlight how to deal with other link functions.

For the log link we find that $v(m(u)) = e^{2u}$, and $m'(u) = e^u$, so that

$$m'(u)v'(m(u)) = 2e^{2u}.$$

Thus,

$$M_{A,\beta_0,\delta}(x) = 2\|x\| \sup\left\{e^{2\beta^\top x} : \|\beta - \beta_0\| \leq \delta\right\},$$

which means our condition on $M_{A,\beta_0,\delta}$ is that X has a finite moment generating function in a neighbourhood of $4\beta_0$. We also take

$$M_{B,\beta_0,\delta}(x) = \|x\|^2 \sup\left\{e^{\beta^\top x} : \|\beta - \beta_0\| \leq \delta\right\},$$

which leads to the strictly weaker condition that X has a finite moment generating function in a neighbourhood of $2\beta_0$. Finally,

$$\frac{(m'(u))^2}{v(m(u))} = 1,$$

whose derivative is 0 so we need only

$$M_{C,\beta_0,\delta}(u) = 0.$$

Thus, $C_n(\beta)$ does not depend on β, so consistency is a consequence of two finite moments for X. The conditions on $v(m(\beta_0^\top X))$ and on $m'(\beta_0^\top X)$ are the same as those above; they amount to a finite moment generating function of X at $4\beta_0$ and at $2\beta_0$.

For the square root link, we have $v(m) = m^2/k$, $m(u) = u^2$, and $v(m(u)) = u^4/k$. We find

$$m'(u)v'(m(u)) = 4u^3/k.$$

Thus,

$$M_{A,\beta_0,\delta}(x) = 4\|x\| \left(|\beta_0^\top x| + \delta\|x\|\right)^3 /k,$$

which means we require X to have 8 finite moments. Evidently, $m''(u) = 2$. Thus, we may take

$$M_{B,\beta_0,\delta}(x) = 2\|x\|^2,$$

which leads to a weaker condition, namely, 4 finite moments for X. Finally,

$$\frac{(m'(u))^2}{v(m(u))} = \frac{4u^2k}{u^4} = \frac{4k}{u^2}.$$

This function diverges at $u = 0$, so we need to add an assumption: there is an $\epsilon > 0$ and a $\delta > 0$ such that, for all β with $\|\beta - \beta_0\| \leq \delta$, we have

$$P(\beta^\top X \geq \epsilon) = 1.$$

In this case,

$$\frac{d}{du}\frac{(m'(u))^2}{v(m(u))} = -8u^3,$$

and $M_{C,\beta_0,\delta}(x) \leq 8\|x\|^3/\epsilon^3$. This amounts to three finite moments for X. The conditions on $v(m(\beta_0^\top X))$ and on $m'(\beta_0^\top X)$ amount to $\mathrm{E}((\beta_0^\top X)^8) < \infty$ and $\mathrm{E}((\beta_0^\top X)^2\|X\|^2) < \infty$, i.e., 8 finite moments which matches the requirement for $M_{A,\beta_0,\delta}$.

Inverse Gaussian Family: Here $v(m) = m^3/\lambda$. For the log link, $m(u) = e^u$, we find that $v(m(u)) = e^{3u}/\lambda$ so that

$$M_{A,\beta_0,\delta}(x) = 3\|x\| \sup\{e^{3\beta^\top x} : \|\beta - \beta_0\| \leq \delta\}/\lambda,$$

which means our condition on $M_{A,\beta_0,\delta}$ is that X has a finite moment generating function in a neighbourhood of $6\beta_0$. We also have

$$m''(u) = m'(u) = e^u,$$

and therefore take

$$M_{B,\beta_0,\delta}(x) = \|x\|^2 \sup\{e^{\beta^\top x} : \|\beta - \beta_0\| \le \delta\},$$

which leads to the strictly weaker condition that X has a finite moment generating function in a neighbourhood of $2\beta_0$. Finally,

$$\frac{(m'(u))^2}{v(m(u))} = \frac{\lambda}{e^u},$$

and so

$$\frac{d}{du}\frac{(m'(u))^2}{v(m(u))} = \frac{d}{du}\frac{\lambda}{e^u} = \frac{-\lambda}{e^u}.$$

Thus,

$$M_{C,\beta_0,\delta}(u) = \lambda \|x\|^3 \cdot \sup\{e^{-\beta^\top x} : \|\beta - \beta_0\| \le \delta\}.$$

Our condition is that X has a finite moment generating function in some neighbourhood of $-\beta_0$. We also need $\mathrm{E}(e^{6\beta_0^\top X}) < \infty$, and $\mathrm{E}(\|X\|^2 e^{2\beta_0^\top X}) < \infty$. Our overall condition is therefore that X has a finite moment generating function in some neighbourhood of $6\beta_0$ and in some neighbourhood of $-\beta_0$. We remark that the set of β where a moment generating function is finite is convex and will include both these neighbourhoods and a tube containing them.

Negative Binomial Family: We have $v(m) = m + m^2/k$, with $k > 0$. We consider the log link, although the square root and identity links are also sometimes used. For the log link, $m(u) = e^u$, and

$$|w_A(u)| = e^u + \frac{2e^{2u}}{k},$$

so that

$$M_{A,\beta_0,\delta}(x) = \|x\| \sup\{e^{\beta^\top x} + 2e^{2\beta^\top x}/k, \|\beta - \beta_0\| \le \delta\}.$$

Our condition then amounts to X having a finite moment generating function in a neighbourhood of $4\beta_0$. Also,

$$M_{B,\beta_0,\delta}(x) = \|x\|^2 \sup\{e^{\beta^\top x}, \|\beta - \beta_0\| \le \delta\},$$

which leads to the strictly weaker condition of X having a finite moment generating function in a neighbourhood of $2\beta_0$. For C_n, we see that

$$\left|\frac{d}{du}\frac{(m'(u))^2}{v(m(u))}\right| = \frac{e^u}{(1 + e^u/k)^2} \le C,$$

for some constant $C > 0$. Therefore, our condition associated with $M_{C,\beta_0,\delta}(x)$ is that X has three finite moments. We also require $\mathrm{E}(v^2(m(\beta_0^\top X))) < \infty$, $\mathrm{E}(e^{2\beta_0^\top X}\|X\|^2) < \infty$, and $\mathrm{E}(\|X\|^2) < \infty$, but these hold, provided that the above conditions on the moment generating function of X are satisfied.

For each row of in Table S.8 we examined the 6 required conditions and identified the most stringent moment assumptions required. These are presented in Table S.9.

Supplementary Table S.9: Moment conditions on X for the consistency of Σ_n. For the Negative Binomial distribution the parameter k is assumed to be known.

Distribution	$m(u)$	$v(m)$	Moment Conditions
Normal(μ, σ^2)	u	1	Covariates have finite second moments
Poisson(λ)	e^u	m	X has a finite MGF in a neighbourhood of $2\beta_0$
Poisson(λ)	u^2	m	Covariates have finite fourth moments
Bernoulli(π)	$F(u)$	$m(1-m)$	Covariates have finite fourth moments
Gamma(μ, k)	e^u	m^2	X has a finite MGF in a neighbourhood of $4\beta_0$, **and** the covariates have finite second moments
IG(μ, λ)	e^u	m^3	X has a finite MGF in neighbourhoods of $6\beta_0$ and $-\beta_0$
NB(μ, k)	e^u	$m + m^2/k$	X has a finite MGF in a neighbourhood of $4\beta_0$, **and** the covariates have finite third moments

S.2.2.3 Condition (C)

This condition requires that $\beta_0^\top X$ have a continuous distribution. In particular, it will not be satisfied with only discrete covariates or with $\beta_0 = 0$. This is a real restriction; if the covariates are unrelated to the response then in the limit all of the observations will be in a single cell.

S.2.3 Condition (D) and the rank of the covariance

In Theorem S.2 we gave conditions under which condition (D1) holds for the particular estimator $\Sigma_n(\beta_n)$. In our discussion below we show that under somewhat stronger conditions on the choices of cell boundaries, and on the distribution of the covariates, we have rank$(\Sigma) \in \{G, G-1\}$, and identify conditions on the link and variance functions that determine which of the two possibilities is correct. We also establish that these stronger conditions imply condition (D2), that rank$(\Sigma_n) \xrightarrow{p}$ rank(Σ). More generally, however, condition (D2) can be more difficult to verify. Our simulation results from Section 5 suggest that the verification of condition (D2) should not be a major concern. Nevertheless, we provide an alternative approach that can be taken to avoid this potential problem.

Along the lines of Proposition 2 of Lütkepohl and Burda (1997) and the "trimmed" or "Winsorized" tests of Davidov et al. (2018), the main idea is to make use of the eigendecompositions of Σ_n and Σ, so that $\Sigma_n = E_n \Lambda_n E_n^\top$ and $\Sigma = E \Lambda E^\top$, where the columns of E, E_n are orthogonal, and Λ, Λ_n are diagonal matrices. Then, we can "trim" Σ_n by setting all entries of Λ_n that are smaller than some $c > 0$ to zero. This prevents undesirable instabilities when making use of generalized inverses of Σ_n in test statistics. We refer readers to Lütkepohl and Burda (1997) and Davidov et al. (2018) for more information. We have not needed to use this suggestion in our simulations.

S.2.3.1 Condition (D2)

In this subsection we present results on the rank of $\Sigma(\beta)$ and on the limit of the rank of $\Sigma_n(\tilde{\beta}_n)$ when the sequence of random parameter values $\tilde{\beta}_n$ converges to some β^*.

S.2.3.2 Rank of the covariance matrix

Note that the rank of $\Sigma(\beta, \phi)$ does not depend on $\phi > 0$, so we take $\phi = 1$ and drop the symbol from our notation.

The rank in question depends on how many of the cells have positive probability for the distribution of $\beta^\top X$. It is certainly possible to have distributions for X and cell boundaries k_g such that

$$P(k_{g-1} < \beta^\top X \leq k_g) = 0$$

for one or more values of g. If this happens, then such a cell could be combined with an adjacent cell without changing the law of our test statistic. We will compute the rank under the assumption that β, the law of X, and the cell boundaries are related in such a way that there is, in the limit, no chance of any empty cells.

The rank of $\Sigma(\beta)$ may depend on whether or not our model has an intercept. We now introduce notation to allow us to talk about both cases. In models *with an intercept* we assume that the first column of the design matrix X^* is a column of 1s. Then there are d columns corresponding to a d-dimensional covariate X. In models *without an intercept* there is no column of 1s, just d columns of the covariate X. The column of 1s is distinguished from other columns of X because of the assumption made below that X has a Lebesgue density.

Our discussion will be simpler if we temporarily let ξ be the vector of coefficients of the random covariates X and α be the intercept if one is present. Then, $\beta = \xi$ if there is no intercept and $\beta = (\alpha, \xi^\top)^\top$ if there is an intercept. The linear predictor is correspondingly either $\eta(X) = \xi^\top X$ or $\eta(X) = \alpha + \xi^\top X$. In this section and again when we study consistency of our test in Subsection S.2.4 we will repeatedly use the dependence of $\eta(X)$ on β.

We now consider a parameter vector β satisfying the following condition:

Condition (C*(β))

The X_i are i.i.d. with a joint density, say g_X. The vector β lies in the interior of $\mathbf{B}_0$. There is an $\epsilon > 0$ such that the density of the linear predictor, $\eta(X)$, is positive almost everywhere on the interval

$$L_\epsilon \equiv [k_1 - \epsilon, k_{G-1} + \epsilon].$$

To compute the rank we study the quadratic form

$$\mathbf{a}^\top \Sigma(\beta) \mathbf{a},$$

for $\mathbf{a} = (a_1, \ldots, a_G)^\top \in \mathbb{R}^G$. The rank is G minus the dimension of the vector subspace consisting of those $\mathbf{a}$ for which the quadratic form is 0. We have the following theorem.

Theorem S.3. *Let $\mathbf{a} = (a_1, \ldots, a_G)^\top \in \mathbb{R}^G$ and fix a vector β such that the basic moment conditions (S.6), (S.7), and (S.8) all hold. Assume that v, m, and m' are continuous functions. Under condition C*(β) we have the following conclusions:*

1. *If the entries a_g are not all equal then*
$$\mathbf{a}^\top \Sigma(\beta) \mathbf{a} > 0.$$

2. *If the entries a_g are all equal and non-zero and our model has an intercept then*
$$\mathbf{a}^\top \Sigma(\beta) \mathbf{a} > 0$$
unless there are constants b and c such that
$$v(m(u)) = m'(u)(b + cu) \tag{S.9}$$
for all u in the support of $\eta(X)$.

3. *If the entries a_g are all equal and non-zero and our model does not have an intercept then*
$$\mathbf{a}^\top \Sigma(\beta) \mathbf{a} > 0$$
unless there is a constant c such that
$$v(m(u)) = m'(u)cu \tag{S.10}$$
for all u in the support of $\eta(X)$.

4. *If the entries a_g are all equal, our model has an intercept and there are b and c such that (S.9) holds for all u in the support of $\eta(X)$ then*
$$\mathbf{a}^\top \Sigma(\beta) \mathbf{a} = 0.$$

5. *If the entries a_g are all equal, our model does not have an intercept and there is a c such that (S.10) holds for all u in the support of $\eta(X)$ then*
$$\mathbf{a}^\top \Sigma(\beta) \mathbf{a} = 0.$$

Thus, under our condition C*(β), the rank of $\Sigma(\beta)$ is G unless the conditions of parts (4) or (5) of Theorem S.3 hold, in which case the rank is $G - 1$.

Proof of Theorem S.3: In this proof we usually suppress the dependence of objects on β; it is useful to remember that $\eta(X)$ depends on β.

The quadratic forms in the theorem of the form $\mathbf{a}^\top \Sigma \mathbf{a}$ and $\mathbf{a}^\top \Sigma_n \mathbf{a}$ arise from the residuals in a linear regression problem. To be specific, from a data point X we define a response variable $L(\mathbf{a}, X)$ by

$$L(\mathbf{a}, X) = \sum_g a_g \mathbb{1}\left(k_{g-1} < \eta(X) \leq k_g\right) v^{1/2}(m(\eta(X))).$$

Let $X_c = X$ if we have no intercept and let $X_c = (1, X^\top)^\top$ of we do have an intercept. Our predictor is $w^{1/2}(X) X_c$, where

$$w^{1/2}(X) = \frac{m'(\eta(X))}{v^{1/2}(m(\eta(X)))}.$$

Thus we fit, with β fixed, the model

$$L(\mathbf{a}, X) = w^{1/2}(X) X_c^\top \gamma_c + \Xi,$$

where Ξ is a notional error and $\gamma_c = (\gamma_0, \gamma^\top)^\top$ is a $d+1$ vector to be fitted if the model has an intercept and $\gamma_c = \gamma$ is a d vector if the model does not have an intercept. The mean squared error when $L(\mathbf{a}, X)$ is predicted by $w^{1/2}(X) X_c^\top \gamma_c$ is

$$\text{MSE}(\gamma_c) \equiv \mathrm{E}\left\{ [L(\mathbf{a}, X) - w^{1/2}(X) X_c^\top \gamma_c]^2 \right\}.$$

Since the weight w is positive everywhere, this function of γ_c is quadratic and has a positive minimum unless there is a γ_c such that

$$P\left(L(\mathbf{a}, X) = w^{1/2}(X) \gamma_c^\top X_c \right) = 1. \tag{S.11}$$

Expanding out and simplifying we see that the mean square error at the given γ_c is

$$\text{MSE}(\gamma_c) = \mathbf{a}^\top \Sigma^{(1)}(\beta) \mathbf{a} - 2\mathbf{a}^\top \Delta \gamma_c + \gamma_c^\top I(\beta) \gamma_c.$$

This quantity is minimized over γ_c by the choice

$$\gamma_c = I^{-1}(\beta) \Delta \mathbf{a}.$$

At this choice of γ_c the MSE simplifies to

$$\mathbf{a}^\top \left\{ \Sigma^{(1)} - \Delta I^{-1}(\beta) \Delta^\top \right\} \mathbf{a} = \mathbf{a}^\top \Sigma(\beta) \mathbf{a}.$$

We now prove that if the a_g are not all the same number then the probability in (S.11) is less than 1 and that if the a_g are all equal then this probability is less than 1 unless the appropriate identity holds for all u in the support of η for some c and, in the case of models with an intercept, some b.

Fix a γ_c and let M_L denote the set of x for which

$$L(\mathbf{a}, x) \neq w^{1/2}(x)\gamma_c^\top x_c.$$

Suppose first that the a_g are not all equal. Then there is a g^* with $a_{g^*} \neq a_{g^*+1}$. Consider the following functions on the real line:

$$H_1(u) = \sum_g a_g \mathbb{1}\left(k_{g-1} < u \leq k_g\right) v^{1/2}(m(u))$$

and

$$H_2(u) = \frac{m'(u)}{v^{1/2}(m(u))}.$$

Then M_L is the set of x such that

$$\frac{H_1(\eta(x))}{H_2(\eta(x))} \neq \gamma_0 + \gamma^\top x,$$

where we take $\gamma_0 = 0$ if our model has no intercept.

We can always write γ in the form $c\xi + \zeta$ where $\zeta^\top \xi = 0$. Then M_L is the set of x such that

$$\frac{H_1(\eta(x))}{H_2(\eta(x))} \neq \gamma_0 - c\alpha + c(\eta(x)) + \zeta^\top x. \tag{S.12}$$

Without an intercept we drop the term α and recall $\gamma_0 = 0$.

Every term in (S.12) except $\zeta^\top x$ depends on x only through $\xi^\top x$. It follows that, almost surely,

$$\operatorname{Var}(\zeta^\top X \mid \xi^\top X) = 0.$$

If X has a joint density, $\zeta \neq 0$, $\xi \neq 0$, and $\zeta^\top \xi = 0$, then the vector $(\zeta^\top X, \xi^\top X)$ also has a joint density and the indicated conditional variance is almost surely not 0. We deduce $\zeta = 0$. (The existence of the joint density in question can be seen by making a change of variables from the original X variables to OX where O is an orthogonal matrix whose first two rows are $\zeta/\|\zeta\|$ and $\xi/\|\xi\|$. Then, marginalize to the joint density of $\zeta^\top X/\|\zeta\|, \xi^\top X/\|\xi\|$. Finally, make a scale change of variables to see that $(\zeta^\top X, \xi^\top X)$ has a joint density.)

Thus, M_L is the set of x for which

$$\frac{H_1(\eta(x))}{H_2(\eta(x))} \neq \gamma_0 - c\alpha + c\eta(x).$$

The difference between the two sides is $H_3(\eta(x))$, where

$$H_3(u) = \frac{\sum_g a_g \mathbb{1}\left(k_{g-1} < u \leq k_g\right) v(m(u))}{m'(u)} - \gamma_0 + c\alpha - cu.$$

This function of u is discontinuous at $u = k_{g^*}$; it is left continuous but discontinuous from the right at that point. It is continuous on $(k_{g^*} - \delta, k_{g^*}]$ and on the interval $(k_{g^*}, k_{g^*} + \delta)$ for all sufficiently small δ. Choosing $\delta < \epsilon$ from Condition C*(β), we find that there is a $\delta > 0$ so small that M_L contains either

$$\{x : k_{g^*} < \alpha + \beta^\top x \leq k_{g^*} + \delta\}$$

or

$$\{x : k_{g^*} - \delta < \alpha + \beta^\top x \le k_{g^*}\}.$$

Since both of these intervals are in the support of $\eta(X)$ the minimized value of the quadratic form is positive.

We have now proved the first statement of the theorem so that the null space of $\Sigma(\beta)$ must be 0 or the span of the vector $\mathbf{a}$ with all $a_i = 1$. In turn, we see that the rank of $\Sigma(\beta)$ is either G or $G-1$.

To prove the remaining four assertions in the theorem we now assume without loss of generality that $a_g = 1$ for all g. Then, H_1 simplifies to

$$H_1(u) = v^{1/2}(m(u)).$$

The function H_3 above becomes

$$H_3(u) = \frac{v(m(u))}{m'(u)} - \gamma_0 + c\alpha - cu.$$

Let M be the set of $u \in \operatorname{supp}\{\eta(X)\}$ for which $H_3(u) \neq 0$. If there is a γ_c for which M is empty then our vector $\mathbf{a} = (1, \dots, 1)^\top$ is in the null space of $\Sigma(\beta)$ and $\operatorname{rank}(\Sigma(\beta)) = G - 1$. In particular, if there is no intercept and identity (S.10) holds for all $u \in \operatorname{supp}\{\eta(X)\}$, then M is empty. This proves assertion 5 of the theorem. Similarly, if there is an intercept and identity (S.9) holds for all $u \in \operatorname{supp}\{\eta(X)\}$, then M is empty. This proves assertion 4 of the theorem.

Finally, if the set M has positive Lebesgue measure for every choice of γ_c then $\mathbf{a} = (1, \dots, 1)^\top$ is not in the null space and we have $\operatorname{rank}(\Sigma(\beta)) = G$. These are assertions 2 and 3 of the theorem.

This concludes the proof of Theorem S.3.

Remark 1. *The relevant identities hold in some important special cases:*

1. *We are fitting an intercept in a Gaussian model and take $v(m)$ constant and m the identity function.*

2. *We are fitting a Poisson regression model with the square root link so that $m(u) = u^2$ and $v(m) = m$. Since (S.10) holds, we get rank $G-1$ whether or not we are fitting an intercept.*

3. *We are fitting a Poisson regression model with the log link so that $m(u) = e^u$ and $v(m) = m$. Since (S.10) holds, we get rank $G-1$ whether or not we are fitting an intercept.*

S.2.3.3 Rank of the covariance estimator

We now turn to the rank of the estimate $\Sigma_n(\tilde\beta_n)$ where $\tilde\beta_n$ is a sequence of possibly random parameter vectors. We want to give conditions under which

$$\operatorname{rank}(\Sigma_n(\tilde\beta_n)) \xrightarrow{p} \operatorname{rank}(\Sigma(\beta)). \tag{S.13}$$

We will need to strengthen condition (C*(β)) to make it uniform in a neighbourhood of β.

Condition (C*(β, δ))

The X_i are i.i.d. with a joint density, say g_X. There is a $\epsilon > 0$ such that for every β' such that $\|\beta' - \beta\| \leq \delta$ the density of the linear predictor, $\eta(X, \beta')$, is positive almost everywhere on the interval

$$L_\epsilon \equiv [k_1 - \epsilon, k_{G-1} + \epsilon].$$

Then, we will need to add conditions to guarantee that Σ is continuous in a neighbourhood of β; those will be condition (A) and condition (E(β)). These imply

$$\Sigma_n(\tilde{\beta}_n) \xrightarrow{p} \Sigma(\beta);$$

see Theorem S.2. Unless one of the identities (S.9) or (S.10) holds (on the support of $\eta(X)$), the rank of $\Sigma(\beta)$ is G under our conditions. Since Σ_n converges to $\Sigma(\beta)$ and $\beta^* \in \mathbf{B}_G$, we find that Σ_n has rank G for large n in this case.

Next, we consider models in which one of our identities does hold for all u.

Theorem S.4. *Fix a parameter vector β. Assume that the possibly random interval endpoints $k_{n,g}$ converge in probability to non-random limits k_g that are distinct. Assume that condition (C*(β, δ)) holds for some $\delta > 0$. Assume condition (E(β)). Let $\tilde{\beta}_n$ be a sequence of possibly random parameter vectors converging in probability to β. Then, $\Sigma_n(\tilde{\beta}_n) \xrightarrow{p} \Sigma(\beta)$. Moreover:*

1. *If our model has an intercept and for every pair of reals (b, c)*

$$P\{v(m(\eta(X))) = m'(\eta(X))(b + c\eta(X))\} < 1, \tag{S.14}$$

then

$$\text{rank}(\Sigma_n(\tilde{\beta}_n)) \xrightarrow{p} \text{rank}(\Sigma(\beta)) = G.$$

2. *If our model has an intercept and there is a pair of reals (b, c) such that*

$$P\{v(m(\eta(X))) = m'(\eta(X))(b + c\eta(X))\} = 1, \tag{S.15}$$

then

$$\text{rank}(\Sigma_n(\tilde{\beta}_n)) \xrightarrow{p} \text{rank}(\Sigma(\beta)) = G - 1.$$

3. *If our model does not have an intercept and for every real c*

$$P\{v(m(\eta(X))) = m'(\eta(X))c\eta(X)\} < 1, \tag{S.16}$$

then

$$\text{rank}(\Sigma_n(\tilde{\beta}_n)) \xrightarrow{p} \text{rank}(\Sigma(\beta)) = G.$$

4. If our model does not have an intercept and there is a real c such that

$$P\left\{v(m(\eta(X))) = m'(\eta(X))c\eta(X)\right\} = 1, \tag{S.17}$$

then

$$\text{rank}(\Sigma_n(\tilde{\beta}_n)) \xrightarrow{p} \text{rank}(\Sigma(\beta)) = G - 1.$$

Proof of Theorem S.4: Theorem S.3 shows that the ranks asserted for $\Sigma(\beta)$ are correct. The same theorem guarantees that $\Sigma(\beta')$ is continuous in β' in some δ neighbourhood of β and that $\Sigma_n(\beta')$ to $\Sigma(\beta')$ uniformly for β' in that δ neighbourhood of β. It follows that $\Sigma_n(\tilde{\beta}_n) \xrightarrow{p} \Sigma(\beta)$.

If a sequence of square $G \times S$ matrices M_n converges to a matrix M then

$$\liminf \text{rank}(M_n) \geq \text{rank}(M).$$

If $\text{rank}(M) = G$ this means

$$\lim \text{rank}(M_n) = G.$$

Applied to the sequence $\Sigma_n(\tilde{\beta}_n)$ with limit $\Sigma(\beta)$ we learn that

$$\text{rank}(M_n) \xrightarrow{p} G.$$

This proves the first and third enumerated assertions. Under the conditions of the second and fourth assertions we need only identify some $\mathbf{a}$ such that

$$P\left\{\mathbf{a}^\top \Sigma_n(\beta_n)\mathbf{a} = 0\right\} \to 1.$$

We show this holds for $\mathbf{a}$ with all $a_g = 1$. The matrix in question multiplied by n is the error sum of squares in a regression. Let $\tilde{\eta}_n(X)$ be the linear predictor using $\tilde{\beta}_n$. The response vector is the n vector with ith entry

$$\sum_g a_g \mathbb{1}\left(k_{n,g-1} < \tilde{\eta}_n(X_i) \leq k_{n,g}\right) v^{1/2}(m(\tilde{\eta}_n(X_i))) = v^{1/2}(m(\tilde{\eta}_n(X_i))).$$

If there is an intercept in the model the predictor matrix is the $n \times (d+1)$ matrix with ith row

$$\frac{m'(\tilde{\eta}_n(X_i))}{v^{1/2}(m(\eta_n(X_i)))}(1, X_i^\top).$$

If there is no intercept in the model, drop the 1. If we multiply the matrix of predictors on the right by the column vector $\tilde{\beta}$ we get a column vector with ith entry

$$\frac{m'(\tilde{\eta}_n(X_i))}{v^{1/2}(m(\tilde{\eta}_n(X_i)))}\tilde{\eta}_n(X_i).$$

If there is no intercept the relevant identity guarantees that after multiplying by some non-zero c this is exactly the response vector. Thus

$$\operatorname{rank}(\Sigma_n(\tilde{\beta}_n)) \le G - 1.$$

The fourth assertion follows.

The argument for the second assertion is only a bit more difficult. This concludes the proof of Theorem S.4.

S.2.4 Consistency of our test

In this section we discuss power in terms of consistency; we outline one possible set of conditions on the alternative distribution, the specific model, and the choice of cell boundaries that will ensure that our test is consistent. We generalize Theorem 3 of the main paper and prove the generalization. In order to do so we will need to extend our discussion of ranks.

Our conclusions are affected by the presence or absence of an intercept term. Again, our linear predictor is $\eta(X) = \alpha + \xi^\top X$ if there is an intercept and $\eta(X) = \xi^\top X$ if there is no interecpt. We use β for the complete parameter vector which we define as $\beta = (\alpha, \xi^\top)^\top$ if we have an intercept and just ξ if not.

S.2.4.1 Behaviour of coefficient estimator under the alternative

Our first set of assumptions (in which all expectations are computed under the alternative) are used to verify the conditions in White (1982), which guarantee that the estimate β_n has a limit under the alternative being considered; we denote this limit by β^* and use the corresponding notation α^* and ξ^* in order to differentiate between models with and without an intercept.

Condition (K1)

The following all hold:

1. $\operatorname{Var}(Y) < \infty$.

2. The model does not have an unknown dispersion parameter. (This assumption can easily be weakened to the existence of a non-zero limit of the sequence of estimates of the dispersion parameter.)

3. The model fitting exercise is restricted to $\{\beta \in \mathbf{B}\}$ for some specified compact subset $\mathbf{B}$ of $\mathbb{R}^d$ or $\mathbb{R}^{d+1}$ with a non-empty interior contained in $\mathbf{B}_0$. The parameter space $\mathbf{B}$ (for the regression parameters) is a subset of $\mathbb{R}^{d+1}$ if we fit an intercept and $\mathbb{R}^d$ if not.

4. If the model does not have an intercept then we have $\mathbf{0} \notin \mathbf{B}$. If the model has an intercept then for every α we have $(\alpha, \mathbf{0}) \notin \mathbf{B}$.

5. For all $\beta \in \mathbf{B}$ the model mean function is square integrable, that is,

$$\mathrm{E}(m^2(\beta^\top X)) < \infty.$$

6. The Fisher information matrix $I_1(\beta)$ is defined and positive definite for all $\beta \in \mathbf{B}$ and depends continuously on β over $\mathbf{B}$.

7. Condition (E(β)) holds for all $\beta \in \mathbf{B}$.

8. The (X_i, Y_i) are i.i.d. with a joint density g.

The conditions above guarantee that a number of the assumptions in White (1982) hold. These assumptions are enough to ensure the existence of a (possibly not unique) maximizer, over $\mathbf{B}$, of the GLM likelihood. We now add further assumptions, also taken from White (1982), to guarantee that this maximizer is unique and that our estimator, β_n, converges to the unique value, which we call $\beta*$.

Condition (K2)

The following hold:

1.

$$\mathrm{E}(|\log g(X,Y)|) < \infty,$$

and there is a random variable Z_{KL} such that for all $\beta \in \mathbf{B}$,

$$|Y \cdot (b')^{-1}\left\{m(\beta^\top X_c)\right\} - b\left((b')^{-1}\left\{m(\beta^\top X_c)\right\}\right)| \leq Z_{\mathrm{KL}}$$

and

$$\mathrm{E}\left\{Z_{\mathrm{KL}}\right\} < \infty.$$

2. The Kullback-Leibler divergence,

$$\mathrm{KL}(g : f, \beta) = \mathrm{E} \log\left[g(X,Y)/f(X,Y,\beta)\right],$$

has a unique minimizer over $\beta \in \mathbf{B}$, denoted β^*.

Note that whether the second assumption in (K2) holds depends on the true alternative; we follow White (1982) and simply add this assumption of uniqueness. Our conditions (K1) and (K2) now imply Assumptions A1, A2, and A3 of White (1982). In turn, these imply almost sure convergence of β_n to $\beta^* \in \mathbf{B}$. Let $\eta^*(X)$ be the linear predictor evaluated at β^*, that is, $\eta^*(X) = \alpha^* + \xi^{*\top} X$ if our model has an intercept and $\eta^*(X) = \xi^{*\top} X$ if not.

S.2.4.2 Behaviour of interval endpoints under the alternative

In our discussion of the null distribution we considered both fixed and random interval endpoints. In our consistency results we need assumptions about the probability that $\eta(X)$ belongs to each of the limiting intervals; these probabilities depend on β and will be false for any β with $\xi = 0$. (This motivates (K1.4).) If the support of $\eta(X)$ (which can depend on which β is considered) is bounded, then for some choices of intervals there will be intervals with no observations. We need to assume that this does not happen for the predictor η^*. The following condition strengthens condition (C).

Condition (K3)

The following hold:

1. Under the alternative, the interval endpoints $k_{n,g}$ converge in probability to some limit values k_g. The k_g are all distinct.

2. Condition C*(β) holds with $\beta = \beta^*$. That is, if $\mathbf{B}_G$ is defined to be the set of $\beta \in \mathbf{B}$ such that there is an $\epsilon > 0$ for which the density of the linear predictor, $\eta(X)$, is positive on the interval

$$L_\epsilon \equiv [k_1 - \epsilon, k_{G-1} + \epsilon],$$

then the limit β^* is in $\mathbf{B}_G$.

Under condition (K3), no vector β with $\xi = 0$ is in $\mathbf{B}_G$. Therefore, at a minimum we are assuming $\xi^* \neq 0$. For other β it is possible that the support of η is bounded; in that case some methods of choosing boundaries (like fixed boundaries) may eliminate that β from $\mathbf{B}_G$. In our simulations we have chosen cell boundaries using the estimate β_n so as to make all the cells have approximately the same sum of variances of the responses.

S.2.4.3 Behaviour of covariance estimator under the alternative

Next, we consider our estimate Σ_n of Σ. Condition (E(β_0)) implies that our estimate Σ_n is consistent for $\Sigma(\beta_0)$ under the null hypothesis. We extend these conditions to every $\beta \in \mathbf{B}$ so that they apply to the unknown value β^*. Specifically, we assume

Condition (K4)

Condition (E(β)) holds for every $\beta \in \mathbf{B}$.

We now show that under reasonable conditions (including the assumption that $\beta \in \mathbf{B}_G$) the matrix $\Sigma(\beta)$ has rank G or $G-1$; when n is large $\Sigma_n(\beta)$ has the same rank with high probability. Rank $G-1$ arises only in some special cases that we will describe. Under our conditions these ranks are the same for all $\beta \in \mathbf{B}_G$. Our next theorem is an easy consequence of Theorem S.4.

Theorem S.5. *Assume conditions (K1), (K3), and (K4). Then,*

$$\Sigma_n(\beta_n) \xrightarrow{p} \Sigma(\beta^*),$$

and

$$\operatorname{rank}\{\Sigma_n(\beta_n)\} \xrightarrow{p} \operatorname{rank}(\Sigma(\beta^*)).$$

S.2.4.4 Consistency

Our consistency result requires that we have modeled the mean incorrectly in a fairly strong sense. For $1 \le g \le G$, define

$$\mu_g(\beta) = \mathrm{E}\{\mathbb{1}(k_{g-1} < \eta(X) \le k_g) m(\eta(X))\}$$

and

$$\mu_{g,A}(\beta) = \mathrm{E}\{\mathbb{1}(k_{g-1} < \eta(X) \le k_g) Y\}.$$

Also define

$$\bar{\mu}(\beta) = \frac{1}{G}\sum_{g=1}^{G} \mu_g(\beta)$$

and

$$\bar{\mu}_A(\beta) = \frac{1}{G}\sum_{g=1}^{G} \mu_{g,A}(\beta).$$

Condition (K5)

One of the following holds:

1. The model fitted does not have an intercept, the set of u in the support of $\beta^{*\top} X$ where the identity (S.10) does not hold has positive Lebesgue measure for every choice of c, and for all $\beta \in \mathbf{B}$

$$\sum_g (\mu_g(\beta) - \mu_{g,A}(\beta))^2 > 0.$$

2. The model fitted does not have an intercept, the identity (S.10) holds for all u, and for all $\beta \in \mathbf{B}$

$$\sum_g (\mu_g(\beta) - \mu_{g,A}(\beta) - \bar{\mu}(\beta) + \bar{\mu}_A(\beta))^2 > 0.$$

3. The model fitted has an intercept, the set of u in the support of $\alpha^* + \beta^{*\top} X$ where the identity (S.9) does not hold has positive Lebesgue measure for every choice of b and c, and for all $\beta \in \mathbf{B}$ we have

$$\sum_g (\mu_g(\beta) - \mu_{g,A}(\beta))^2 > 0.$$

4. The model fitted has an intercept, the identity (S.9) holds for all u, and for all $\beta \in \mathbf{B}$ we have

$$\sum_g \left(\mu_g(\beta) - \mu_{g,A}(\beta) - \bar{\mu}(\beta) + \bar{\mu}_A(\beta)\right)^2 > 0.$$

We now restate and prove Theorem 3 from the main paper.

Theorem S.6. *Under conditions (K1), (K2), (K3), (K4), and (K5),*

$$X^2_{GHL} \xrightarrow{p} \infty,$$

and the test based on X^2_{GHL} is consistent against the alternative in question.

Proof of Theorem 3: Suppose first that (K5.1) or (K5.3) holds. Then, $\text{rank}(\Sigma(\beta^*)) = G$. Our statistic is at least as large as

$$\lambda_{\min}(\Sigma_n^{-1}) S_n^{1\top} S_n^1 = \frac{1}{\lambda_{\max}(\Sigma_n)} S_n^{1\top} S_n^1,$$

where $\lambda_{\min}(M)$ denotes the smallest eigenvalue of the matrix M. The eigenvalue $\lambda_{\max}(\Sigma_n)$ has a non-zero limit so our claim is that

$$S_n^{1\top} S_n^1 \xrightarrow{p} \infty.$$

Clearly, it is enough to show that

$$\frac{1}{n} S_n^{1\top} S_n^1 \xrightarrow{p} \sum_g \left(\mu_g(\beta) - \mu_{g,A}(\beta)\right)^2,$$

in probability, since we have assumed the indicated limit is positive.

Our proof is much like the proof of consistency of $\sigma_n(\beta_n)$. Let $\delta > 0$ be a value for which $E(\beta^*)$ holds and let $\kappa \in (0, \delta)$. Let $\mathcal{N}_\kappa = \{\beta : \|\beta - \beta^*\| \le \kappa\}$. Define

$$h(X, Y, \beta) = Y - m(\beta^\top X).$$

Apply Lemma 2 with this function h, with X, Y playing the role of X and $K = N_\kappa$. This function h satisfies conditions (i) and (ii) of Lemma 2 in view of (K.1) and (E(β^*)). Then, Lemma 2 shows that the family $\mathcal{F}_{h,K}$ is a Glivenko-Cantelli class. In particular,

$$W_n(u, \beta) \equiv \frac{1}{n} \sum_{i=1}^n \left\{Y_i - m(\beta^\top X)\right\} \mathbb{1}(\beta^\top X_i \le u)$$

converges almost surely, uniformly over $(u, \beta) \in \mathbb{R} \times N_\kappa$ to its expectation, which is

$$\tau(u, \beta) \equiv \mathrm{E}\left\{(m^*(X) - m(\beta^\top X)\right\} \mathbb{1}(\beta^\top X_i \le u).$$

The function $\tau(u, \beta)$ is continuous in both β and u by the dominated convergence theorem (dominating function $(m^*(X))^2 + (M^*(X))^2$ with M^* as given in Lemma 2). This conclusion uses the fact that $\beta^\top X$ has a density for all $\beta \in \mathbf{B}$ to deduce that

$$P(\beta^\top X = u) = 0$$

for all $\beta \in N_\kappa$ and all real u.

The gth entry in the vector S_n^1 is

$$\sqrt{n}\left\{W_n(k_{n,g},\beta) - W_n(k_{n,g-1},\beta)\right\}.$$

We deduce that the gth entry in $S_n^1/\sqrt{n}$ converges in probability to

$$\tau(k_g,\beta^*) - \tau(k_{g-1},\beta^*).$$

(The convergence is not guaranteed to be almost sure unless the $k_{n,g}$ converge almost surely to the k_g.) Finally, this convergence implies

$$\frac{1}{n} S_n^{1\top} S_n^1 \xrightarrow{p} \sum_g \left(\mu_g(\beta) - \mu_{g,A}(\beta)\right)^2,$$

in probability, as was needed.

Now suppose (K5.2) or (K5.4) holds. Then except for an event of probability converging to 0 as $n \to \infty$, the rank of Σ_n is $G-1$. When the rank of Σ_n is $G-1$ the matrix Σ_n has $G-1$ non-zero eigenvalues and the matrix Σ_n^+ also has $G-1$ non-zero eigenvalues. Let $\lambda^*_{\min}(\Sigma_n^+)$ denote the smallest non-zero eigenvalue of Σ_n^+. We have

$$\lambda^*_{\min}(\Sigma_n^+) = \frac{1}{\lambda_{\max}(\Sigma_n)}.$$

The remaining eigenvalue is 0 and corresponds in both cases to the eignvector $\mathbf{1}_G$, a column vector of G ones. Let $\mathbf{I}_G$ be the $G \times G$ identity matrix and

$$\mathbf{H}_G = \mathbf{I}_G - \frac{1}{n}\mathbf{1}_G\mathbf{1}_G^\top.$$

Since $\mathbf{1}_G$ is in the null space of Σ_n, we have

$$S_n^{1\top}\Sigma_n^+ S_n^1 = \left(\mathbf{H}_G S_n^1\right)^\top \Sigma_n^+ \left(\mathbf{H}_G S_n^1\right) \geq \frac{1}{\lambda_{\max}(\Sigma_n)} S_n^{1\top}\mathbf{H}_G S_n^1.$$

An argument similar to that for the previous case shows that

$$\frac{1}{n} S_n^{1\top}\mathbf{h}_G S_n^1 \xrightarrow{p} \sum_g \left(\mu_g(\beta) - \mu_{g,A}(\beta) - \bar{\mu}(\beta) + \bar{\mu}_A\right)^2.$$

Again, we see that

$$S_n^{1\top} S_n^1 \xrightarrow{p} \infty.$$

Consistency is an immediate consequence of this limit and the existence of the limit law given in Theorem 1 of the main text.

S.2.5 Empirical Process Lemmas

The proofs above used two lemmas, which in turn depend on a third. All of the lemmas synthesize results in Kosorok (2007).

Lemma 1. *Suppose $X_1, X_2, \ldots$ are i.i.d. d-dimensional vectors with the same distribution as X, and β is a d-dimensional vector taking values in some open set O. Suppose h is a real-valued function of the pair (x, β), which is continuously differentiable in β for each x. Let K be some compact subset of O with diameter denoted by diam(K). Assume:*

1. *there is some $\beta^* \in K$ such that*
$$\mathrm{E}\left\{|h(X, \beta^*)|\right\} < \infty,$$
2. *there is a function $M(x)$ such that for all x in the support of X and all $\beta \in K$*
$$\left\| \frac{\partial}{\partial \beta} h(x, \beta) \right\| \leq M(x),$$
and,
3. *the random variable $M(X)$ is integrable:*
$$\mathrm{E}(M(X)) < \infty.$$

Then,

1. *The family of functions*
$$\mathcal{F}_{h,K} = \{x \mapsto h(\beta, x); \beta \in K\}$$
is pointwise measurable.
2. *This family has bounded uniform entropy integral with respect to the integrable envelope*
$$M^*(x) = \{M(x) + \mathrm{diam}(K)|h(\beta^*, x)|.$$
3. *The class $\mathcal{F}_{h,K}$ is P-Glivenko-Cantelli, that is,*
$$\sup_{\beta \in K, u \in \mathbb{R}^d} \left| \frac{1}{n} \sum_{i=1}^{n} h(X_i, \beta) - \mathrm{E}\left\{h(X, \beta)\right\} \right| \to 0$$
almost surely.
4. *The map*
$$\beta \mapsto \mathrm{E}\left\{h(X, \beta)\right\}$$
is uniformly continuous on K.

Proof:

To prove the first statement we must find a countable subset $\mathcal{G}$ of $\mathcal{F}_{h,K}$ such that every $f \in \mathcal{F}_{h,K}$ is the pointwise limit of a sequence of elements of $\mathcal{G}$. We find $\mathcal{G}$ by a route which helps with our proof below of the second and third statements.

A set of closed balls of radius δ covers a set $B \subset O$ if B is contained in the union. The covering number of B, denoted by $N(\delta, B, \|\cdot\|)$ is the smallest integer N for which there is a set $\beta_1, \ldots, \beta_N$ of elements of B such that for every $\beta \in B$ there is a j with $\|\beta - \beta_j\| \le \delta$. The set of such balls is said to δ-cover B. We now bound, by a standard volume argument, the value of N when B is a ball.

The volume of a ball of radius r in $\mathbb{R}^d$ is proportional to r^d. Thus if there are N disjoint balls of radius ϵ in $\mathbb{R}^d$ all of which lie in some ball of radius R then the volume of those N small balls is N times the volume of a single one and less than the volume of the ball of radius R. So $N \le (R/\epsilon)^d$. Consider now a set of such balls of maximal size; this collection is said to pack B and the corresponding value of N is the ϵ packing number. The collection of N balls with the same centers but radius 2ϵ contains the ball of radius R for if not we could fit in another ball of radius ϵ. So for any ball B of radius R we get

$$N(\epsilon, B, \|\cdot\|) \le (2R/\epsilon)^d.$$

For each $\epsilon > 0$ we have identified a finite set say B_ϵ of points in K such that every point in K is within ϵ of some member β of B_ϵ. Take B to be the union over positive integers n of $B_{1/n}$ and let $\mathcal{G}$ be the corresponding elements of $\mathcal{F}_{j,K}$. Evidently B and $\mathcal{G}$ are countable and B is dense in K. Every $\beta \in K$ is thus the limit of a sequence of points $\beta_n \in B$ and the corresponding f_β is the pointwise limit of f_{β_n} because h is continuous in β. This proves Statement 1.

Now we turn to the second statement. Take B to be a ball of radius $R \le \text{diam}(K)$ which contains K. Find $\beta_1, \ldots, \beta_N$ in B so that $N = N(\epsilon, B, \|\cdot\|)$ and the N balls centered at the β_j having radius ϵ cover B. For each such ball which intersects K let β_j^* be in the intersection. Every point in K is within the union of the balls centered at those β_j for which the intersection with K is not empty. So every point in K is within balls centred at β_j^* but with radius 2ϵ. Thus,

$$N(\epsilon, K, \|\cdot\|) \le (4\text{diam}(K)/\epsilon)^2.$$

Now suppose that $\beta \in K$. Fix $\epsilon > 0$ and find $N = N(\epsilon, K, \|\cdot\|)$ points, say $\{\beta_1, \ldots, \beta_N\}$, in K such that K is contained in union of the N balls of radius ϵ centered at the β_j. For each $f \in \mathcal{F}_{h,K}$ we have $f = f_\beta$ for some $\beta \in K$. Find j so that $\|\beta - \beta_j\| \le \epsilon$. If Q is a finite discrete measure on the support of X there is a set of points $x_1, \ldots, x_k$ and corresponding probabilities $q_1, \ldots, q_k$ such that $\sum_1^k q_i = 1$ and $Q(X = x_i) = q_i$

for $j = 1, \ldots, k$. Now

$$\left|f_\beta(x_i) - f_{\beta_j}(x_i)\right| \le \|\beta - \beta_j\| M(x_i),$$

by Taylor's theorem. Thus, the $L_1(Q)$ norm of $f_\beta - f_{\beta_j}$ satisfies

$$\begin{aligned}
\|f_\beta - f_{\beta_j}\|_{Q,1} &= \sum_{i=1}^{k} |f_\beta(x_i) - f_{\beta_j}(x_i)| q_i \\
&\le \|\beta - \beta_j\| \sum_{i=1}^{k} |M(x_i)| q_i \\
&\le \epsilon \sum_{i=1}^{k} |M^*(x_i)| q_i \\
&\le \epsilon \|M^*\|_{Q,1}.
\end{aligned}$$

Increasing the first argument in the covering number cannot increase the covering number itself. Thus,

$$N\left(\epsilon \|M^*\|_{Q,1}, \mathcal{F}, L_1(Q)\right) \le N\left(\epsilon, K, \|\cdot\|\right) \le (4\mathrm{diam}(K)/\epsilon)^2 < \infty.$$

This proves the bound on the uniform covering numbers

$$\sup_Q N(\epsilon \|M\|_{Q,1}, \mathcal{F}, L_1(Q)) \le N(\epsilon, K, \|\cdot\|) \le (4\mathrm{diam}(K)/\epsilon)^2 < \infty. \tag{S.18}$$

This is Statement 2.

Since pointwise measurability implies P-measurability for every P, we have verified all the conditions of Theorem 8.14, page 145 in Kosorok (2007). Statement 3 follows.

The fourth assertion of the lemma is an application of the Dominated convergence theorem. If the sequence β_n converges to some $\beta \in K$ then $|f_{\beta_n}(x) - f_\beta(x)| < \sup_n \|\beta_n - \beta\| M(x)$ and the right hand side of this inequality is integrable. Uniform continuity is automatic because K is compact. (Indeed, the assumptions on M guarantee that this expectation is a differentiable function of β on the interior of K.)

Our second Lemma deals with processes involving indicators. It deduces Glivenko-Cantelli results from Donsker results; it seems likely that this contributes to an increase in the strength of our moment conditions.

Lemma 2. *Suppose $X_1, X_2, \ldots$ are i.i.d. d-dimensional vectors with the same distribution as X, and β is a d-dimensional vector taking values in some open set O. Suppose h is a real-valued function of the pair (x, β), which is continuously differentiable in β for each x. Let K be some compact subset of O with diameter denoted by diam(K). Assume:*

i) there is some $\beta^* \in K$ such that

$$\mathrm{E}\left\{h^2(X, \beta^*)\right\} < \infty,$$

ii) there is a function $M(x)$ such that for all x in the support of X and all $\beta \in K$

$$\left\| \frac{\partial}{\partial \beta} h(x, \beta) \right\| \leq M(x),$$

and,

iii) the random variable $M(X)$ is square integrable:

$$\mathrm{E}\left(M^2(X)\right) < \infty.$$

Then,

1. *The class of functions*

$$\mathcal{F}_{h,K} = \{f_\beta : f_\beta(x) = h(\beta, x), \beta \in K\}$$

has square integrable envelope

$$M^*(x) = \sqrt{2}\sqrt{\{\mathrm{diam}(K)M(x)\}^2 + h^2(\beta^*, x)}.$$

2. *The class of functions $\mathcal{F}_{h,K}$ is pointwise measurable.*

3. *The family of functions*

$$\mathcal{F}_I \equiv \{x \mapsto 1(\beta^\top x \leq u), \beta \in \mathbb{R}^d, u \in \mathbb{R}\} \tag{S.19}$$

is P-measurable for any P and has Vapnik-Chervonenkis dimension $d+2$. This family has bounded uniform entropy integral with envelope 1.

4. *The family*

$$\mathcal{F}_A = \{f : f = f_1 f_2, f_1 \in \mathcal{F}_I, f_2 \in \mathcal{F}_{h,K}\}$$

has bounded uniform entropy integral with respect to the envelope M^*. This envelope is square integrable.

5. *The family $\mathcal{F}_A$ is P-measurable for any P. For each $0 < \delta \leq \infty$ the family*

$$\mathcal{F}_{A,\delta} = \left\{f - g : f, g \in \mathcal{F}_A, \mathrm{E}\left\{f(X) - g(X)\right\}^2 < \delta^2\right\}$$

is P-measurable for any P. The family

$$\mathcal{F}^2_{A,\infty} = \left\{(f-g)^2 : f, g \in \mathcal{F}_A\right\}$$

is P measurable for all P.

6. *The family $\mathcal{F}_A$ is P-Donsker.*

7. *The family* $\mathcal{F}_{h,K}$ *is* P*-Donsker.*

8. *The family* $\mathcal{F}_{h,K}$ *is* P*-Glivenko-Cantelli.*

Proof:

The first statement is elementary. The second statement is contained in Lemma 1. The third statement is Lemma 9.12 on page 161 of Kosorok (2007).

The fourth statement is a consequence of Theorem 9.15 of Kosorok (2007) which asserts that given two classes $\mathcal{F}_1$ and $\mathcal{F}_2$ which have bounded entropy integral with envelopes M_1 and M_2 the class of all products of a function from each has a bounded uniform entropy integral with envelope the product $M_1 M_2$.

The fifth statement follows from a small extension of Lemma 8.12 on page 143 in Kosorok (2007); see Lemma 3 below.

The sixth statement is a consequence of the fourth and fifth and Theorem 8.19 on page 149 in Kosorok (2007). Any subfamily of a P-Donsker family is P-Donsker; the seventh assertion follows. The final statement is the assertion that P-Donsker implies P-Glivenko-Cantelli which is contained in Lemma 8.17 on page 148.

Lemma 3. *Let* $\mathcal{G}$ *be a pointwise measurable family of functions on* $\mathbb{R}^d$ *and let* $\mathcal{F}_I$ *be the family in (S.19). Then, the family*

$$\mathcal{H} \equiv \mathcal{G}\mathcal{F}_I \equiv \{fg : f \in \mathcal{F}_I, g \in \mathcal{G}\}$$

is P*-measurable for all* P*. Moreover, if we define for* $0 < \delta < \infty$ *the class*

$$\mathcal{H}_\delta = \left\{ f - g : f, g \in \mathcal{H}, \mathrm{E}\left\{f(X) - g(X)\right\}^2 < \delta^2 \right\}$$

and the class

$$\mathcal{H}^2_\infty = \left\{ (f-g)^2 : f, g \in \mathcal{H} \right\},$$

then all these classes are P*-measurable for any* P*.*

The proof of the Lemma is entirely analogous to that of Lemma 8.12 on page 143 in Kosorok (2007).

References

Donald W.K. Andrews. Asymptotic results for generalized Wald tests. *Econometric Theory*, 3(3):348–358, 1987.

Christopher R. Bilder and Thomas M. Loughin. *Analysis of Categorical Data with R.* Chapman and Hall/CRC, 2014.

Ori Davidov, Casey M. Jelsema, and Shyamal Peddada. Testing for inequality constraints in singular models by trimming or Winsorizing the variance matrix. *Journal of the American Statistical Association*, 113 (522):906–918, 2018.

Tracy DeHart, Howard Tennen, Stephen Armeli, Michael Todd, and Glenn Affleck. Drinking to regulate negative romantic relationship interactions: The moderating role of self-esteem. *Journal of Experimental Social Psychology*, 44(3):527–538, 2008.

Ludwig Fahrmeir and Heinz Kaufmann. Consistency and asymptotic normality of the maximum likelihood estimator in generalized linear models. *The Annals of Statistics*, pages 342–368, 1985.

Ludwig Fahrmeir and Heinz Kaufmann. Correction: consistency and asymptotic normality of the maximum likelihood estimator in generalized linear models. *The Annals of Statistics*, 14(4):1643–1643, 1986.

John P. Klein and Melvin L. Moeschberger. *Survival Analysis: Techniques for Censored and Truncated Data*. Springer Science & Business Media, 1997.

Michael R. Kosorok. *Introduction to Empirical Processes and Semiparametric Inference*. Springer Series in Statistics. Springer New York, 2007.

Helmut Lütkepohl and Maike M. Burda. Modified Wald tests under nonregular conditions. *Journal of Econometrics*, 78(2):315–332, 1997.

Winfried Stute. Nonparametric model checks for regression. *The Annals of Statistics*, pages 613–641, 1997.

Winfried Stute and Li-Xing Zhu. Model checks for generalized linear models. *Scandinavian Journal of Statistics*, 29(3):535–545, 2002.

Winfried Stute, Silke Thies, and Li-Xing Zhu. Model checks for regression: an innovation process approach. *The Annals of Statistics*, 26(5):1916–1934, 1998.

John Q. Su and Lee-Jen Wei. A lack-of-fit test for the mean function in a generalized linear model. *Journal of the American Statistical Association*, 86(414):420–426, 1991.

Halbert White. Maximum likelihood estimation of misspecified models. *Econometrica*, 50(1):1–25, 1982.
